# Mechanisms of the Action of Fire-Retardants on Reducing the Flammability of Certain Classes of Polymers and Glass-Reinforced Plastics Based on the Study of Their Combustion

**DOI:** 10.3390/polym14214523

**Published:** 2022-10-26

**Authors:** Oleg Korobeinichev, Andrey Shmakov, Alexander Paletsky, Stanislav Trubachev, Artem Shaklein, Alexander Karpov, Egor Sosnin, Sergey Kostritsa, Amit Kumar, Vladimir Shvartsberg

**Affiliations:** 1 Voevodsky Institute of Chemical Kinetics and Combustion SB RAS, 630090 Novosibirsk, Russia; 2 Udmurt Federal Research Center, 426067 Izhevsk, Russia; 3 Department of Physics, Novosibirsk State University, 630090 Novosibirsk, Russia; 4 P.I. Baranov Central Institute of Aviation Motor Development, 111116 Moscow, Russia; 5 Department of Aerospace Engineering, Indian Institute of Technology Madras, Chennai 600036, India

**Keywords:** polymer composites, flame-retardants, flame spread, numerical modeling, flame structure, inhibition mechanism, pyrolysis, flammability, opposed flow, counterflow

## Abstract

In the present review, using an integrated approach based on the experimental and theoretical study of the processes of thermal decomposition and combustion of practically important polymers, such as polymethyl methacrylate, polyethylene, and glass-fiber-reinforced epoxy resin, the features of the mechanism for reducing the combustibility of these materials with phosphorus-containing flame-retardants (FR), as well as graphene, are identified. A set of original experimental methods was developed and applied that make it possible to study the kinetics of thermal decomposition and the thermal and chemical structure of the flames of the studied materials, including those with FR additives, as well as to measure the flame propagation velocity, the mass burning rate, and the heat fluxes from the flame on the surface of a material. Numerical models were developed and tested to describe the key parameters of the flames of the studied polymeric materials. An analysis of the experimental and numerical simulation data presented showed that the main effect of phosphorus-containing fire-retardants on reducing the combustibility of these materials is associated with the inhibition of combustion processes in the gas phase, and the effect of adding graphene manifests itself in both gas and condensed phases.

## 1. Introduction

At present, the use of polymeric materials is constantly growing: they are widely used in the building industry, automotives, railways, air transportation, shipbuilding, the electric power industry, etc. The use of polymers as constructional materials provides good strength and weight characteristics, and as ornamental materials, they ensure necessary comfort and opportunities for design. In recent years, the greatest growth in the use of polymeric materials has been noted for constructional materials, where many elements are made from polymer composites. However, polymeric materials, unlike most metals, are capable of exothermic oxidation reactions and have a fire hazard (they ignite, spread flame, emit heat, smoke, and give off the toxic products of thermal-oxidative degradation). Therefore, one of the urgent tasks researchers and engineers around the world are trying to solve is a way to reduce the fire hazard of polymeric materials and the structural elements based on them.

Currently, work to reduce the fire hazard of polymeric materials is mainly carried out in two main directions: (1) increasing the thermal stability and reducing the heat of the combustion of the polymeric component; (2) the use of various flame-retardants.

The main method of searching for flame-retardants that reduce the flammability of polymers and reinforced plastics is to carry out standard tests for their combustibility, such as LOI, UL-94, TGA, cone calorimetry, etc. At the same time, in some cases, there is no correlation between the effectiveness of individual flame-retardants in composite materials according to individual tests. For example, some fire-retardants may be more active in LOI tests, while others may be more active in UL-94 tests.

There is also a problem of predicting the behavior of polymeric materials under conditions different from the conditions of standard tests for combustibility, since, in the latter, it is impossible to take into account all the conditions that arise in a real situation during a fire, for example, the existence of powerful thermal radiation fluxes from the flame to the material surface, the turbulence of gas flows, etc. In addition, the problem of optimizing and reducing expensive tests is also relevant. These problems stimulate both experimental and theoretical research and the development of mathematical models that describe the response of combustibles material to fire conditions.

In recent decades, numerous important works have appeared in the literature, devoted to the experimental study of the processes of pyrolysis, ignition, and combustion of various polymers, including those with various flame-retardants additives. In these works, mainly the polymers important for practical use—such as polyethylene, polypropylene, polyvinyl chloride, polyurethane, polymethyl methacrylate, polystyrene, polybutylene terephthalate, epoxy resin, etc., which are widely used in the electric power industry, construction, transportation, and other fields—were investigated as the objects of study. Polyethylene and polyvinyl chloride, widely used electrical insulation materials for electrical cables and wires, have been studied to determine the ignition conditions and the flame propagation characteristics of these products [1,2,3]. In these studies, original experimental setups were used, which allowed researchers to obtain more information and parameters for the processes of pyrolysis, ignition, and combustion than in the case of standard tests. For modern, thermally insulating materials used for construction and transport, such as polyurethane foam and expanded polystyrene, the effects of composition, morphology, material sample sizes, and pressure on the rate of the formation and composition of pyrolysis products, as well as the combustibility of these polymers, were researched [4,5,6,7,8,9]. Huang et al. developed an original experimental setup and used it to study the effect of expanded graphite, aluminum hypophosphite, and aluminum diethylhypophosphite additives on the flammability of rigid polyurethane foam [10]. In a study on the combustibility of thermally insulating materials, such as expanded polystyrene, researchers [11,12,13] developed and applied original experimental methods and theoretical models of the processes of flame propagation over this polymer, which were previously absent in the literature. In a number of works, the influence of the flame-retardant additives of various types of action on the formation of hazardous combustion products and on the combustibility of polymers such as polybutylene terephthalate, as well as composite materials based on epoxy resins, which are in high demand for air transport, was investigated [14,15,16]. In [17], the influence of phosphorus-containing flame-retardants such as FR 1025 and Exolit OP 1230 on the combustibility of polybutylene terephthalate under the influence of an external heat flux was studied. Using mass spectrometry with photoionization from VUV radiation, Ma et al. studied the mechanism of action of additives of various phosphorus-containing flame-retardants, such as aluminum hypophosphite (AHP) and aluminum diethylphosphinate (AlPi), on the pyrolysis of polybutylene terephthalate [18]. Using PLIF and thermal analysis methods, Geschwindner et al. studied the effect of pentaerythritol spirobis (methylphosphonate) on the combustion and thermal decomposition of polypropylene [19].

It should be noted that, among a large number of abovementioned polymers and composites, polymethyl methacrylate (PMMA), polyethylene (PE), and glass-fiber-reinforced epoxy resin (GFRER) hold the most unique positions. PMMA is a thermoplastic polymer that has become widely used due to a number of unique properties, such as transparency and strength; it is used as a finishing material and a glaze in the building industry and in the manufacturing of aircraft and ship windows. Polyethylene is one of the most common manmade polymer materials in the world today, and it is a thermoplastic polymer that has high chemical resistance and electrical insulating properties; hence, it is used in the manufacturing of a wide variety of products, ranging from packaging bags, facade building panels, and ending with electrical insulation for wires and high-voltage cables. Reinforced plastics based on epoxy resins, due to their high strength and technological effectiveness, are widely used in aviation, shipbuilding, railway transportation, the electric power industry, and other industries.

The modern approach to improving the thermal stability of polymers and reinforced plastics consists of introducing halogen-free flame-retardants into their composition [20]. The advantage of phosphorus-containing flame-retardants, such as triphenyl phosphate (TPP) [21], 9,10-dehydro,9-oxa,10-phosphophenanthrene,10-oxide (DOPO) [22], and red phosphorus, is that they are supposed to affect the combustibility of materials both through the formation of a carbon frame in the condensed phase and through the gas phase mechanism [20,21].

There are several ways to improve the thermal stability of acrylic plastics by adding flame-retardants, for example, adding flame-retardants during PMMA hot pressing [23] and copolymerizing them with MMA [24,25,26]. When obtaining PMMA with reduced flammability, special attention should be paid to maintaining its strength and transparency. In particular, a small concentration of Cloisite 93A in the composition of PMMA nanocomposites slightly worsens the light transmission index in the visible and ultraviolet ranges, reducing the rate of thermal decomposition of the composite and increasing the yield of the carbon residue [25]. Nanocomposites of PMMA with TPP, Cloisite 93A, and their combinations demonstrate better thermal stability than PMMA without additives: Flame-retardants reduce the peak heat release rate and the total heat release in tests using a cone calorimeter. It has been found that TPP can form a protective polyphosphate film on the surface. The main mechanisms for improving the thermal stability and fire resistance of PMMA nanocomposites are the barrier effects of Cloisite 93A nanoclay, an increase in the mass of the carbon skeleton, an increase in melt viscosity, and gas-phase flame inhibition due to trapping radicals. Cross-linking long polymer chains to form three-dimensional networks is an effective method of improving the mechanical and thermal properties of polymers [27,28]. Phosphorus-containing monomer synthesized in [29] was introduced into PMMA with radical copolymerization. With a 15% phosphorus-containing monomer, the copolymer exhibited comparable transparency to PMMA, passed the UL-94 V-0 test, and had an oxygen index (LOI) of 27.5%. In [23], the effect of DOPO flame-retardant on the flammability of PMMA using Fourier IR spectroscopy, thermogravimetric analysis (TGA), and other methods was investigated. The DOPO additive reacts with unsaturated bonds at the end of the PMMA polymer chain during extrusion and/or injection molding to form phosphorus–carbon bonds. As the flame-retardant content increased, the glass transition temperature and pyrolysis rate decreased. The authors concluded that the flame-retardant acts in the condensed phase. In [30], it was shown that the copolymerization of MMA with phosphorus–chlorine-containing dimethacrylate and 3-methacryl oxypropyl trimethoxysilane modifier leads to a significant decrease in the combustibility of the PMMA composite, with an increase in the oxygen index from 18% to 30.5%. A synergistic effect between nanosized alumina and silicon oxides and ammonium polyphosphate (APP) flame-retardant was identified for PMMA and polystyrene in [31]. With the total addition of a fire-retardant of 15%, it was possible to achieve an increase in the oxygen index from 18 to 23% for the modified PMMA. With the total addition of a fire-retardant of 15%, it was possible to achieve an increase in the oxygen index from 18 to 23% for the modified PMMA. The thermal decomposition of a mixture of flame-retardant additives showed a strong interaction between alumina and silicon oxides and phosphorus-containing substances, which led to a change in the decomposition pathway of APP. Furthermore, the addition of a fire-retardant led to the formation of a protective carbon layer on the PMMA surface.

There are numerous works in the literature on the study of the effect of phosphorus-containing flame-retardants on the combustibility of various types of polyethylene. The majority of these works are related to evaluating the flammability reduction of blends of polyethylene with flame-retardant additives using standard methods, such as LOI measurement, burning tests (UL94), the cone calorimeter test, and TGA and DSC analyses.

In the work of Peters [32], based on TGA, DSC, and LOI measurements, the effect of adding red phosphorus (RP) on the flammability of high-density polyethylene (HDPE) was studied. Adding 12% wt. RP to HDPE leads to an increase in LOI from 18.6% to 21.7%. Analyzing the composition of gaseous combustion products showed that about 98% of the initial amount of RP passes into the gas phase. Thus, in this work, it was experimentally shown that the mechanism for reducing the flammability of HDPE by adding RP is associated with the release of phosphorus oxides into the gas phase. At the same time, the results of the thermal analysis showed that the addition of 8% RP to HDPE increases the thermal stability of HDPE and thereby reduces the yield of the volatile products of HDPE pyrolysis, which are a source of heat during a fire.

The effect of adding RP to HDPE was also investigated using the UL94 VB (vertical burning) test and a cone calorimeter in [33]. A noticeable effect of adding microencapsulated red phosphorus (MRP) to polyethylene was also shown in [34], in which the thermal degradation of the HDPE and MRP composite was studied using thermogravimetric (TG) data obtained at different heating rates. The authors showed the activation energy of pyrolysis for the MRP + HDPE composite was 4% higher than for HDPE. MRP could improve thermal stability and slow down the thermal degradation of HDPE. Wang et al. [35] investigated the synergistic effect of low-density polyethylene (LDPE) between MRP and aluminum hypophosphite (AHP) using LOI, UL-94 VB, and TGA. When the contents of MRP and AHP were 10% and 30%, the LOI of the LDPE/10MRP/30AHP composite was 25.5%, and it passed the UL-94 V-0 rating. The results of cone calorimetry and TGA testing showed that the heat release rate of the composites was significantly reduced, and the thermal stability of the composite was enhanced.

Similar results were also obtained for LDPE composites loading aluminum hydroxide (ATH), RP, and expandable graphite (EG) [36]. The LOI value of LDPE significantly increased from 17.1% to 25.4% upon the incorporation of a 15 wt.% mixture of three fillers with an ATH:RP:EG mass ratio of 1:1:1; this composite achieved the V-0 classification of the UL94 vertical burning test. The TG analysis of this composite under an air atmosphere revealed the thermal stability of the composite to be improved.

It should be noted that the use of RP additives for polymers as flame-retardants has some disadvantages, such as the non-uniform distribution of RP in the composite, the combustibility of RP, etc. Some organic and inorganic phosphorus compounds can be free of the above disadvantages, so they have also been comprehensively studied. For example, in [37], it was found that the addition of 10% triphenyl phosphate (TPP) to the HDPE-based composition led to an increase in LOI above 21%, which also, according to TGA, significantly increased the thermal stability of the mixture. In [38], an EG-g-DOPO complex was synthesized based on 9,10-dihydro-9-oxa-10-phosphaphenanthrene-10-oxide (DOPO) and EG. The results showed that an ultra-high-molecular-weight polyethylene (UHMWPE) composite with 20 wt.% EG-g-DOPO possesses a satisfactory UL-94 flame-retardant grade (V-0) and a high LOI (30.6%).

Thus, triphenyl phosphate, DOPO, and other organophosphorus compounds reduce the flammability of polyethylene due to the effect of these flame-retardants on both the condensed and gas phases.

Phosphorus-containing flame-retardants, such as ammonium polyphosphate (APP), aluminum diethylphosphinate (AlPi), and some others, obviously exhibit a barrier effect, since they contain low-volatility components that form a strong protective layer on the burning surface. Nevertheless, these flame-retardants also show fairly high activity, for example, by increasing the thermal stability of the polymer. Luyt et al. [39] studied the effect of adding a mixture of ammonium polyphosphate (APP) and triazine derivative (TD) (1:3) to LDPE. With a total load of 35 wt.%, the APP + TD was found to be the most efficient for the LDPE, achieving a UL 94 V-0 rating and upgrading the thermal stability of the polymer.

Lau and Atakan [40] studied the thermal decomposition of aluminum diethyl phosphinate (AlPi) as an FR, mixed in UHMWPE using molecular beam mass spectrometry (MBMS) in an oxygen-free atmosphere. It was found that the main product of AlPi is diethylphosphinic acid, which subsequently degrades into lighter species or dimerizes. In the mixture, although the AlPi decomposition is influenced by the polymer in the condensed phase, most of the species responsible for a flame suppressant effect are still present in the gas phase. In the study by [41], the thermal and chemical structures of the diffusion flames of UHMWPE + AlPi specimens were investigated using micro thermocouples and molecular beam mass spectrometry, respectively. In the flames, the concentration of the phosphorus-containing compounds peaked at low heights above the polymer surface, indicating the gas-phase activity of AlPi or its pyrolysis products. Furthermore, a charring layer on top of the burning surface was observed. The use of AlPi as an FR for UHMWPE shows flame-retardant effects in both the condensed and gas phases.

In addition to experimental studies on the effect of phosphorus-containing flame-retardants, there are works in which, by using numerical models, attempts are made to describe the effect of additives of flame-retardants on the combustibility of polymers in standard tests, such as TGA and cone calorimeter tests. In [42], a modeling framework to study the combustion behavior, pyrolysis kinetics, and flame-retardant (FR) mechanisms of FR-treated polymer composites was proposed. The modeling framework satisfactorily predicted the heat release rate (HRR) profiles, ignition time, and combustion duration of the HDPE and HDPE/APP composites, where average relative errors of less than 15% were achieved compared with TGA and cone calorimeter tests.

As can be seen from the above works, the combustion and flame structure of polyethylene with the addition of phosphorus-containing compounds have not been studied in sufficient detail. Thus, in many cases, this does not make it possible to conclude in which phase (in the condensed or gas phase) the effect of a flame-retardant is dominant.

Reinforced polymer composites have high strength, flexibility, chemical resistance, and thermal insulation properties, which, coupled with low density, makes them promising materials for aviation, mechanical engineering, the building industry, etc. The addition of flame-retardants is the main method for reducing their flammability. As a rule, the combustibility of reinforced composites is studied based on standard tests, for example, TG analysis, the cone calorimeter test, UL-94, LOI, FTIR, and mechanical property tests [43,44,45]. There are practically no data in the literature on the effect of flame-retardants on the combustion of reinforced composite materials. In one study, polymer-reinforced natural linen fabric composites, with the addition of 30% APP, self-extinguished within 10 s after the flame was removed in a UL-94 test (V-0), and the LOI increased from 21.3% to 30.3% [46]. APP acts in the condensed phase to create a protective carbonaceous layer on the surface of the composite to prevent combustion. The synthesis of a slow-burning composite material based on carbon foam and epoxy resin achieved the UL-94 V-0 rating in [47], as compared with conventional epoxy. A significant increase in fire resistance is associated with the formation of a carbon layer that protects the polymer from the action of the flame. Oligomeric fire-retardants containing DOPP and DOPI were proposed for commercial epoxy resin RTM6 and carbon-fiber-reinforced RTM6-CF in [48]. Combustibility was significantly reduced due to the inhibition of the combustion processes and char formation in RTM6 + DOPP and RTM6 + DOPI composites, with the inhibition of gas-phase combustion identified as the main extinguishing mechanism. Flammability, as measured by LOI, was significantly reduced, and the UL 94 test classifications V-1 and V-0, respectively, were obtained. The addition of CF to RTM6 + DOPP and RTM6 + DOPI composites reduced the risk of fire in terms of flame propagation even more than flame-retardant addition alone. In addition, a synergy between CF and flame-retardants was found for RTM6-CF + DOPP and RTM6-CF + DOPI composites based on LOI measurements. The UL 94 test showed a V-0 rating for both materials.

In the works presented in this review, for the first time at the molecular level, studies of the mechanism of action of flame-retardants in the gas phase were carried out by measuring the distribution of concentrations of radicals and short-lived species in polymer flames, which has not been performed before. In addition, for the first time, in our work, a numerical coupled model for the combustion of polymers was developed, which takes into account the effect of the addition of some flame-retardants on the rate of chemical reactions of polymer pyrolysis and the combustion of these products in the gas phase.

The aim of this paper was to review the results of studies on the combustion mechanism of PMMA as the polymer whose combustion has been studied in the most detail, as well as polyethylene of various molecular weights and glass-fiber-reinforced epoxy resin with and without flame-retardant additives, carried out mainly by the authors of the article over the last 10 years. Triphenyl phosphate (TPP), DOPO, and graphene were used as flame-retardants. Typically, tests such as LOI, UL-94, cone calorimetry, etc., were used to evaluate the combustibility of composite materials. Although the use of these methods is an effective way to evaluate the combustibility characteristics of inhibited materials at an early stage of combustion, studying the actual behavior of these materials during flame propagation can reveal the advantages and disadvantages of the additives used in real fires. The number of works on reducing the combustibility of solid combustible materials by studying the mechanism of their combustion is limited. A particular feature of our research involves developing numerical models of combustion and models of the action of flame-retardants, as well as a comparison of the results of the experiments and numerical simulation.

## 2. The Experimental Methods for Studying the Combustion Characteristics, Thermal Decomposition, and the Flame Structure of Polymers without Flame-Retardant Additives and with Flame-Retardant Additives

To study the combustion of polymer and composite samples of various types, such as PMMA, PE, and glass-fiber-reinforced plastics based on epoxy resin, various methods have been developed and applied, described below for each type of polymer and composite studied.

### 2.1. PMMA

Slabs of cast composition PMMA and PMMA + 10% (20%) TPP were studied as model samples. Methods for their preparation are described in [49,50].

#### 2.1.1. Investigation of Flame Structure Using Probe Methods

**Figure 1:**
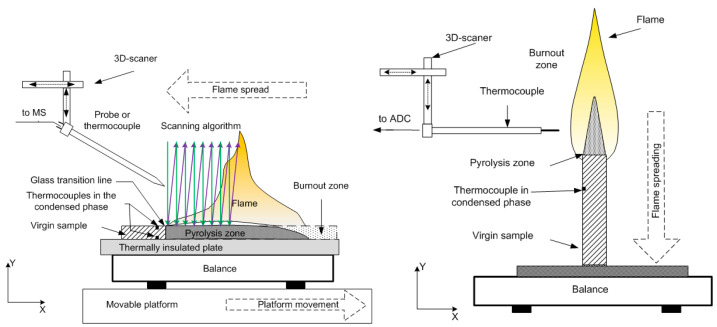
An experimental setup for the study of horizontal (**left**) and vertical (**right**) flame spread over the polymer surface and for measuring temperature and species concentration profiles.

The experimental setups [49,50,51] for horizontal and vertical flame propagation are shown in Figure 1. The slabs were placed in a thin metal frame (0.2 mm thick) to prevent flame propagation along the side surfaces. In the case of flame propagation over a horizontal PMMA surface (Figure 1, left), the sample was mounted on a non-combustible, heat-insulating plate 10 mm thick, which was placed on an electronic balance to determine the mass burnout rate. The plates were ignited from the end with the flame of a propane-butane burner. The rate of the flame spreading over the polymer surface was determined from the video recording of the experiment. The measurement of the flame structure began only after the flame propagation velocity reached a stationary value.

To measure the temperature of the flame, a Pt/Pt + 10%Rh thermocouple [52] was used, made of 50 µm thick wire and coated with a 10 µm thick SiO_2_ layer to prevent catalytic reactions on the thermocouple surface. The thermocouple was installed on a three-coordinate scanning device, and with its help, the thermocouple was moved in space according to a given program (Figure 1). The values of the conductive heat flux from the flame to the solid material for a fixed distance from the flame front were determined with the following formula:

(1) $$q=\lambda \frac{\partial T}{\partial y}$$

where ∂T/∂y is the temperature gradient in the gas phase near the slab surface calculated from experimentally measured temperature profiles, $\lambda =0.5\left(\sum \lambda_{i}X_{i}+{\left(\sum X_{i}/\lambda_{i}\right)}^{-1}\right)$ is the thermal conductivity of the gas mixture [53], and $\lambda_{i}=f\left(T\right)$ is the thermal conductivity of the mixture components [54]. A microprobe with an inlet diameter of 60 microns was mounted on a three-dimensional positioning system. To stabilize the position of the flame front relative to the microprobe, a movable table was used, which, using a stepper motor, moved together with the sample in the direction opposite the direction of the flame propagation at the burning rate. The mechanism for moving the table was turned on after the stationary combustion regime was established. The microprobe was moved at a speed of 2 mm/s from top to bottom (along the *y*-axis—Figure 1) to obtain the vertical mole fractions of the main species in the flame. The molar fractions of MMA, TPP, O_2_, N_2_, CO, CO_2_, and H_2_O in the flame for the studied samples were determined.

**Figure 2:**
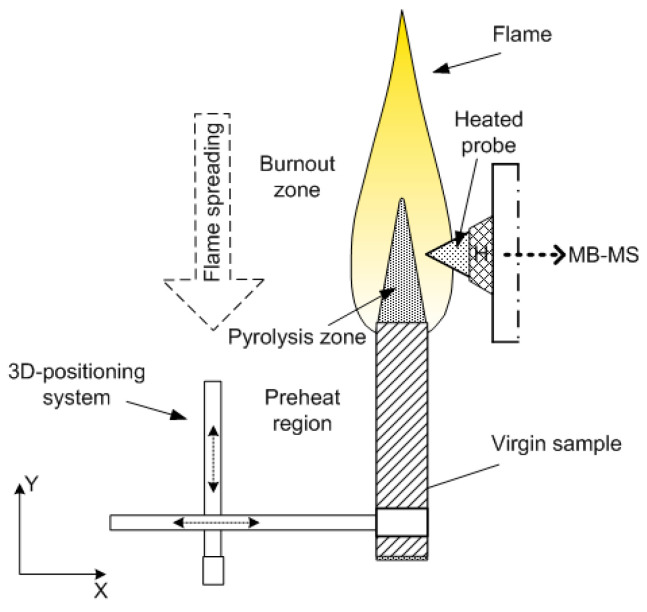
A layout of the experiment for measuring the flame structure using the molecular-beam mass spectrometry (MBMS) method.

The structure of the diffusion flame of a vertically placed 90%PMMA + 10%TPP slab (Figure 2) (4.6 mm thick, 50 mm wide, and 100 mm long) in still air at atmospheric pressure was investigated by the molecular-beam mass spectrometric complex Hiden HPR-60 (the energy of ionizing electrons, 70 eV) [50]. Sampling was conducted in the molecular beam mode with a sonic quartz probe (Figure 1) with an orifice diameter of ~80 µm, an internal angle of 40°, and a cone height of ~10 mm. To prevent TPP and MMA (methyl methacrylate) vapor condensation, the tip of the probe was heated to a temperature of 300–330 °C with an electric heater (Figure 2) [55]. After the stationary sample burning mode was achieved, the sample was moved to the probe at a high velocity with a 3D scanning positioning system.

#### 2.1.2. A Method for Measuring the Fluorescence of the OH Radical in a Flame Spreading over PMMA and PMMA-TPP Using Planar Laser-Induced Fluorescence (PLIF)

**Figure 3:**
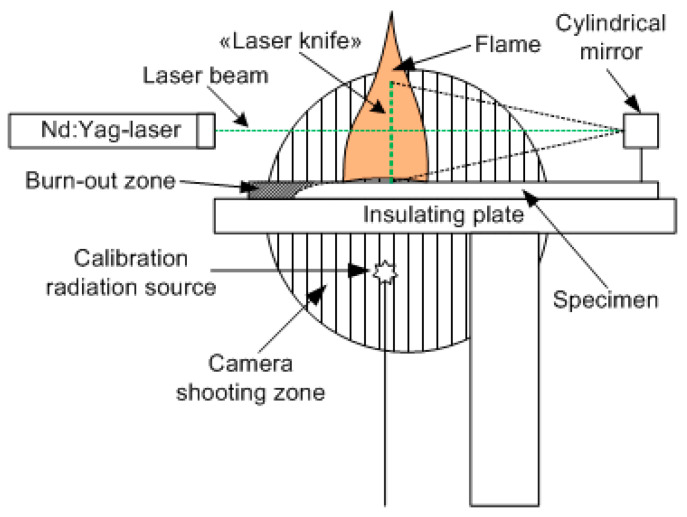
Configuration of the experimental setup.

Figure 3 schematically shows the main components of the experimental setup, as well as the relative position and configuration of the flame and the probe laser beam [56]. The studied samples of cast PMMA and cast PMMA + 10% TPP were inserted into a thin metal frame. The samples were placed horizontally on an asbestos plate. The plane of the laser beam passed along the center line of the slab. The flame spread horizontally in the direction of the cylindrical mirror (Figure 3). The PLIF images were recorded every 30 s in a series of 8 frames with a CCD camera installed perpendicular to the direction of the flame propagation after the burning rate became steady, which was defined in accordance with [49]. OH radicals were excited using a narrow-band tunable dye laser equipped with a system for doubling the frequency of output radiation pumped by the second harmonic of an Nd:YAG laser. The laser radiation wavelength was controlled with a wavelength meter, which enabled the measurement of the wavelength in the visible region with a relative precision equal to 10^−8^. To excite OH radicals, a transition was chosen at a frequency of 31,342.36 cm^−1^ in the band X 2П (0, 0) -> A 2Σ+ (0, 0). The neighboring absorption line, with intensity 10 times less than the selected one, was separated from it by a frequency of 1 cm^−1^. This transition was chosen considering that the population of its lower rotational level was weakly dependent on the temperature. In a temperature range from 1000 to 1400 K, its value will decrease from 3.1 × 10^−17^ to 2.5 × 10^−17^ cm/molecule [57]. The use of a laser with a line width of 0.05 cm^−1^ provides the selective excitation of radicals in this transition and receives LIF signals weakly dependent on the temperature distribution in the flame. A telescope formed by a long-focus spherical lens and a short-focus cylindrical aluminum mirror was used to form a knife-shaped laser beam ~ 100 μm thick and 20 mm high. An image-intensified gated CCD camera, equipped with an ultraviolet objective and a set of short- and long-pass optical filters, was installed perpendicular to the laser propagation plane (in Figure 3, the image capture region is shown as a shaded circle). The image from the excitation region was recorded in a single laser pulse. The laser pulse duration was equal to τ ~ 10 ns, and the fluorescence signal accumulation time was 1 μs. The radiation transmitted through the flame was directed to a diffuser (the calibration radiation source shown in Figure 3). The signal from the diffuser was recorded simultaneously with the signal from the flame. This made it possible to normalize the luminescence signals on the intensity of a single laser pulse and eliminated the influence of fluctuations in the power of a pulsed laser.

### 2.2. PE

The ultra-high-molecular-weight polyethylene (UHMWPE) and polyethylene (PE) specimens were pressed from powder with grain sizes of ∼60, 100, and 80 μm (M.W. ∼2.5 × 10^6^, 10^5^, 3.8 × 10^5^; T_melt_ = 142, 135, and 138 °C) synthesized in the Boreskov Institute of Catalysis (Siberian Branch of Russian Academy of Sciences), together with its mixture with TPP (crystals size ∼40–60 μm; M.W. ∼326; T_melt_ = 40–50 °C; Aldrich, CAS number: 115-86-6). Mixtures of UHMWPE (or PE) + TPP in a ratio of 95/5 and 90/10 (wt%) powders were used in the study and were prepared with mechanical mixing for 15–20 min. UHMWPE (or PE) and UHMWPE (or PE) + TPP (14 mm diam. and 30–40 mm long) specimens were prepared by hot-pressing powders at 140 °C and a pressure of 100 atm. The density of the UHMWPE (PE) specimen was 0.92 g/cm^3^; when 5 wt% TPP was added, the density changed insignificantly to 0.94 g/cm^3^.

#### 2.2.1. The Dynamic Mass Spectrometric Thermal Analysis Method

**Figure 4:**
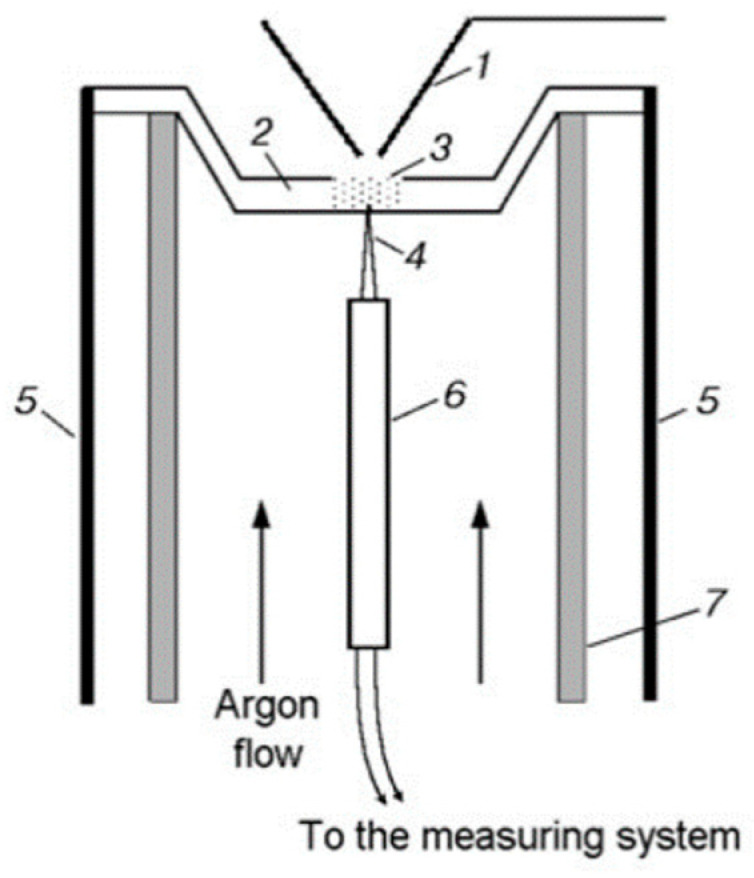
Configuration of the flow reactor: (1) probe; (2) metal cell; (3) UHMWPE (or PE) + TPP powder; (4) thermocouple; (5) electric leads; (6) ceramic tube; (7) quartz tube.

Thermal decomposition under conditions of rapid heating (comparable to the rate of heating in a combustion wave) makes it possible to obtain the most important information about the composition of products near the combustion surface of the condensed system and helps to understand the actual combustion process. Using the method of dynamic mass spectrometric thermal analysis (DMSTA) [58,59] in a flow reactor (Figure 4) in an Ar flow, the thermal decomposition of UHMWPE (or PE) and TPP was studied. The flow reactor was located under the probe of the mass spectrometric complex. The reactor was a quartz tube 1 cm in diameter, in which an argon volumetric flow of ~5 cm^3^/s (n. c.) was created. Inside the tube, there was a metal cell (a crucible of 2 mm deep, 2 mm wide, and 6 mm long) with a total volume of ~0.024 cm^3^, to which a thermocouple was welded in the center from the outside to measure the temperature. The cell was heated by an electric current at a rate of ~150 K/s. A sample of pure polyethylene powder (weighing 1–2 mg) mixed with TPP in a percentage ratio of 95/5 or 90/10% was poured into the cell.

To determine the pyrolysis kinetics of the polymer under study, gaseous reaction products were taken from the flow reactor using a quartz probe. The intensity of peaks in the mass spectrum, which are directly proportional to the rates of formation of the corresponding decomposition products, was measured in the sample, and the temperature of the cell was also measured simultaneously. Based on the time dependences of the rate of product formation and temperature, the Arrhenius dependence of the effective rate constant of the pyrolysis reaction was determined assuming the given reaction order [60].

#### 2.2.2. Measurement of the Diffusion Flame Structure of UHMWPE and PE in Air in the “Candle” Mode

**Figure 5:**
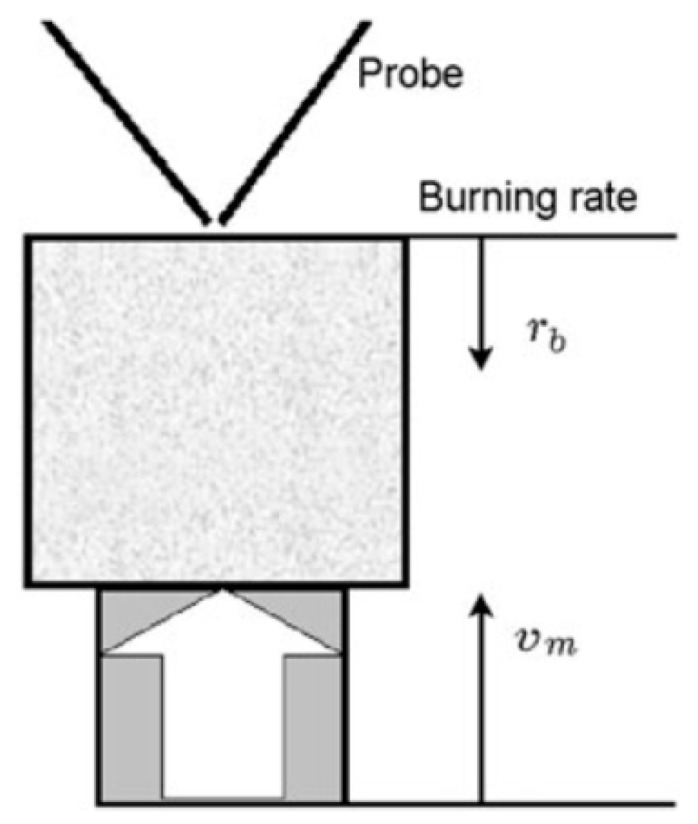
The experimental setup for studying the flame structure.

The structure of the diffusion flame during the combustion of pressed samples of UHMWPE and PE in air was carried out using a sample movement system (Figure 5). The pressed sample was mounted on a holder, and a nichrome heater was placed above the surface of the sample. After ignition, the heater was displaced to the side, and the sample was moved toward the probe by means of a stepper motor at a speed, *V_m_*, exceeding the burning rate of the sample, *r_b_*. Thus, the probe sampled products sequentially from all flame zones [60].

#### 2.2.3. Measuring the Structure of a Diffusion Flame of UHMWPE in Counterflow with Air

**Figure 6:**
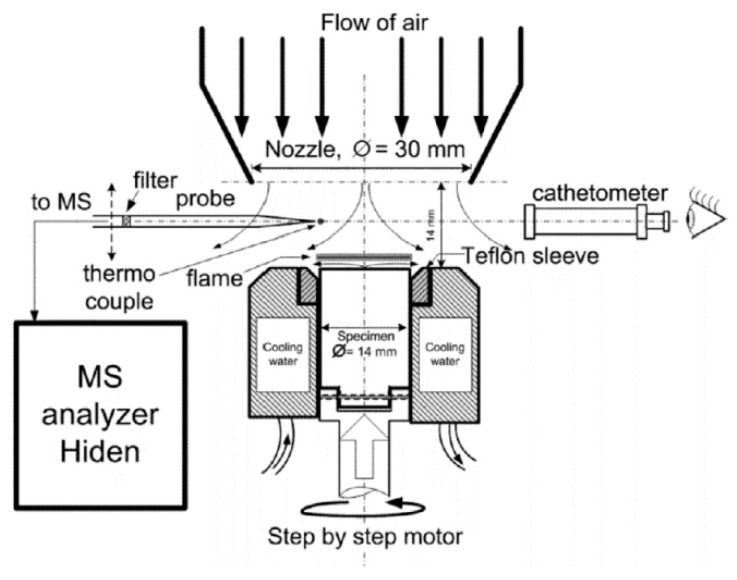
A schematic diagram of the experimental setup for studying the structure of a diffusion flame of UHMWPE in counterflow with air.

The structure of the UHMWPE flame in countercurrent air was measured using a special burner with a polymer feed mechanism and an aerodynamic nozzle, which formed an airflow directed to the top surface of a cylindrical polymer sample [61]. The sample feed mechanism device is shown in Figure 6. During the experiment, the sample rotated around its axis with the help of one stepper motor and simultaneously moved along the burner axis with the help of the second one. During combustion, the samples rotated at a frequency of ~1 Hz inside a thermostatically controlled metal cup at a temperature of 70 °C. The upper part of the sample (~4 mm) was isolated from the walls of the metal shell by a fluoroplastic ring, which reduced the cooling of the UHMWPE melt on the sample surface during combustion. The distance between the nozzle and the sample surface in all experiments was 14 mm; the linear air velocity (under normal conditions) at the nozzle outlet was 43.9 ± 0.13 cm/s. After the samples were ignited with a heater made from nichrome wire, the flame was stabilized in space by moving the rod using a second stepper motor at a fixed speed equal to the burning rate. The movement speed of the sample was determined in special preliminary experiments. A sample from the flame was taken with a quartz microprobe, along with the subsequent injection of the sample into the Hiden HPR60 mass spectrometric complex, as shown in Figure 6.

**Figure 7:**
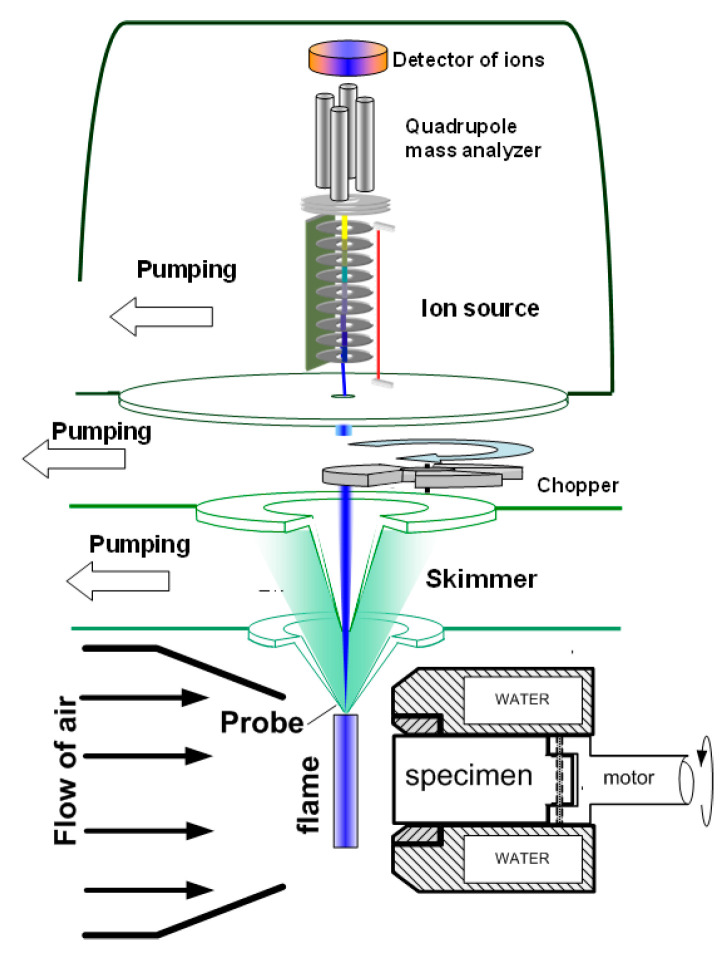
Configuration of the experimental molecular-beam mass spectrometric setup with a “soft” ionization system.

To measure the concentration of species and radicals in a counterflow UHMWPE/air flame, a molecular-beam mass spectrometric setup with a “soft” ionization system was used [62,63]. The experimental setup and its use are shown in Figure 7. The burner was located under the probe in a horizontal position (Figure 7). The probe was installed at a distance of ~1 mm from the edge of the sample. The burner was moved relative to the probe using micro-screws. The motion started from the far zone and went to the surface of the sample. Response factors determined by direct calibration were used to measure the concentration of most species, and for HOPO and HOPO_2_, they were taken from the study by [64], which was previously carried out using this setup, and recalculated in accordance with the used argon ionization energy.

### 2.3. Glass Fiber-Reinforced Epoxy Resin

The GFRER slabs were inserted into a thin aluminum frame (sample holder) 2 mm thick, while the width of the open surface of the sample (over which the flame propagated) was 20 mm. The sample length was 75 mm. The sample and the frame were marked with a step of 10 mm to measure the rate of the flame spread (ROS) from the video recording of the experiments with a FujiFilm x-A20 camcorder (the shooting frequency was 30 frames per second).

**Figure 8:**
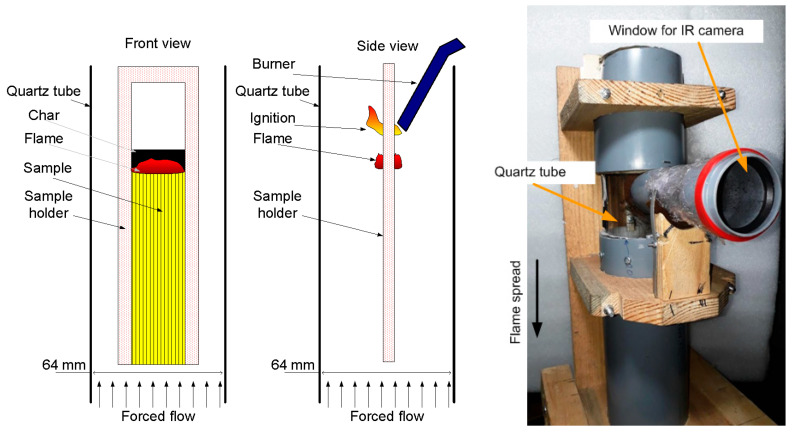
Schematic of the experimental setup for studying the downward flame spread (front view—(**left**); side view—in the (**center**)) and its photo (**right**).

The experimental setup for studying the downward flame spread is shown in Figure 8 [65]. The sample was suspended in a cylindrical transparent quartz tube with a diameter of 64 mm and a length of 45 cm using a duralumin holder. Using MKS flow controllers, a mixture of N_2_ and O_2_ of various concentrations (30–50 vol% O_2_) was fed into the tube through polyethylene hoses. A honeycomb—a foam rubber flow equalizer—was installed in the pipe at the inlet. For all sample types and oxygen concentrations, the flow rate was fixed during the experiment at 4 cm/s. The sample was ignited from above using a propane–butane burner after turning on the opposed oxidizer flow. An opening was made in the pipe, to which a short viewing pipe, 50 mm in diameter, was glued, and it was covered at the end with polyethylene film 0.005 mm thick, passing infrared radiation. Through the viewing pipe, the thermal image of the sample surface during combustion was recorded using an IR camera Guide C400, followed by calculating the temperature on the sample surface as a function of time. The transmission factor of the polyethylene film and the sample surface radiation factor were determined by way of calibration with a Pt-PtRh10% thermocouple, 50 microns thick, embedded into the surface of the same sample. The recording frequency of the IR camera was 1 Hz.

### 2.4. Flammability Tests, TGA, Elemental Analysis

The thermal decomposition properties (TG and DTG) of the samples were measured with a synchronous TG/DTG/DSC analyzer, STA 409 PC (Netzsch) (TGAN), with an aluminum crucible at a flow rate of 27 cm^3^/min (NTP) in air and inert (nitrogen) medium. The mass of the sample was 3–4 mg.

LOI (in accordance with ISO 4589-2) and UL-94 HB (according to EN 60695-11-10) tests were performed according to standard methods. The LOI determination accuracy is ±0.1%. A UL-94 Vertical Bunsen Burner (VBB) test was performed in accordance with ASTM D3801-20a and [66]. Elemental analysis of the quenched samples and soot was carried out using a JEOL JSM-6460LV electron microscope.

## 3. Approaches Developing Numerical Models of Flame Propagation over Solid Combustible Materials with and without Flame-Retardant Additives

### 3.1. Formulation

The following mathematical model was proposed to predict a behavior of flame spread over polymers combined with flame-retardants [49,65,67]:

(2) $$\frac{\partial \rho }{\partial t}+\frac{\partial \rho u_{j}}{\partial x_{j}}=0$$

(3) $$\rho \frac{\partial u_{i}}{\partial t}+\rho u_{j}\frac{\partial u_{i}}{\partial x_{j}}=-\frac{\partial p}{\partial x_{i}}+\frac{\partial }{\partial x_{j}}\mu \frac{\partial u_{i}}{\partial x_{j}}+\left(\rho_{a}-\rho \right)g_{i}$$

(4) $$\rho C\frac{\partial T}{\partial t}+\rho u_{j}C\frac{\partial T}{\partial x_{j}}=\frac{\partial }{\partial x_{j}}\lambda \frac{\partial T}{\partial x_{j}}+\rho WQ-\frac{\partial q_{j}^{r}}{\partial x_{j}}$$

(5) $$\rho \frac{\partial Y_{k}}{\partial t}+\rho u_{j}\frac{\partial Y_{k}}{\partial x_{j}}=\frac{\partial }{\partial x_{j}}\rho D\frac{\partial Y_{k}}{\partial x_{j}}+\nu_{k}\rho W$$

(6) ρ=p/RT

Here $x_{i}=\left\{x,y\right\}$**,** $u_{i}=\left\{u,v\right\}$**,** k={F,O,P}**,** $\nu_{k}=\left\{-1,-\nu_{O},1+\nu_{O}\right\}$**,** $g_{i}=\left\{0,g\right\}$ corresponds to the horizontal orientation of the burning surface of a polymer, $g_{i}=\left\{g,0\right\}$—for a downward flame spread.

Gas phase combustion is modeled by one step reaction in the following way

(7) $$F+\nu_{O}O+I\to \left(1+\nu_{O}\right)P+I$$

The rate of which is calculated as follows:

(8) $$W=kY_{F}Y_{O}\exp\left(-E/R_{0}T\right)$$

The model of solid material was extended to resolve the thermal degradation of the composite material (binder and glass fibers). Thermal conductivity anisotropy resulting from the specific direction of the glass fibers (along the surface) was taken into account. The energy conservation equation of solid material was expressed as:

(9) $$\rho_{s}C_{s}\frac{\partial T_{s}}{\partial t}=\frac{\partial }{\partial x_{j}}\lambda_{s}^{j}\frac{\partial T_{s}}{\partial x_{j}}+\eta_{b}^{0}\rho_{b}Q_{b}W_{b}$$

A reaction rate of pyrolysis reaction is given by:

(10) $$W_{b}={\left(1-\alpha \right)}^{n}k_{b}\exp\left(-E_{b}/R_{0}T_{s}\right)$$

The rate of change of the conversion degree is defined as:

(11) $$\frac{d\alpha }{dt}=W_{b}$$

The solid material density is provided by:

(12) $$\rho_{s}=\eta_{b}^{0}\left(1-\alpha \right)\rho_{b}+\left(1-\eta_{b}^{0}\right)\rho_{f}$$

The mass burning rate is expressed as:

(13) $${\dot{m}}_{b}(x)=\eta_{b}^{0}\rho_{b}\int_{0}^{L_{s}(x)}W_{b}dy$$

According to previous studies [49,64], a DOPO-based flame-retardant is considered to have an effect in the gas phase. Thus, the pre-exponential factor of the gas phase combustion reaction is reduced in the following way:

(14) $$k_{g,DOPO}=\left(1-\Psi_{DOPO}Y_{DOPO}\right)k_{g}$$

where $k_{g}$ is reduced by the factor including the DDM-DOPO mass fraction ($Y_{DOPO}$) and the inhibition effect coefficient ($\Psi_{DOPO}$).

The following approach was designed to take into account an effect of a graphene-based flame-retardant [65], based on the reduction of the combustible part in the release of the total gaseous pyrolysates. Only the $\Psi_{gr}Y_{gr}{\dot{m}}_{b}$ part of the local mass burning rate provided by Equation (13) is set to go to the gaseous fuel (F), while the $\left(1-\Psi_{gr}Y_{gr}\right){\dot{m}}_{b}$ part supplies a non-combustible gas, which was set to be the products (P). Here, $\Psi_{gr}$ represents the inhibition effect coefficient of graphene; $Y_{gr}$ is the graphene mass fraction.

### 3.2. Numerical Method

The set of governing equations is solved by the finite volume method. The PISO algorithm is employed for pressure–velocity coupling. The Euler scheme (first order) was set to approximate time derivatives, the upwind scheme was used for convection terms, and diffusion terms were approximated with the second-order linear scheme. The set of linear algebraic equations was solved by using the conjugate gradient method. The reaction rate of the gas phase combustion reaction was modeled by the Arrhenius form, of which the exponent part produces high nonlinearity for the governing equations. Thus, an iteration procedure integrated in a solution algorithm at each time step was employed here to resolve such obstacles. A solution procedure was implemented in the form of modifications to the OpenFOAM software.

## 4. Combustion Mechanisms of PMMA, PE, and GFRER without Flame-Retardant Additives and with Flame-Retardant Additives

### 4.1. The Gas-Phase Mechanism of Action of TPP on Flame Propagation over PMMA

Table 1 presents the values of the flame propagation rate, mass loss rate, and pyrolysis zone length for PMMA samples without additives and with TPP additives. With the addition of TPP, a noticeable decrease in the mass burning rate and the flame propagation rate was observed. The effect of flame-retardants on the burning rate in relation to the sample without additives is indicated in brackets. Based on all the data obtained, which will be presented below, it was assumed that the reduction in the flame propagation rate caused by TPP additives occurred due to the influence of the flame-retardant on the reaction rate in the gas phase, as well as due to the dilution effect.

**Figure 9:**
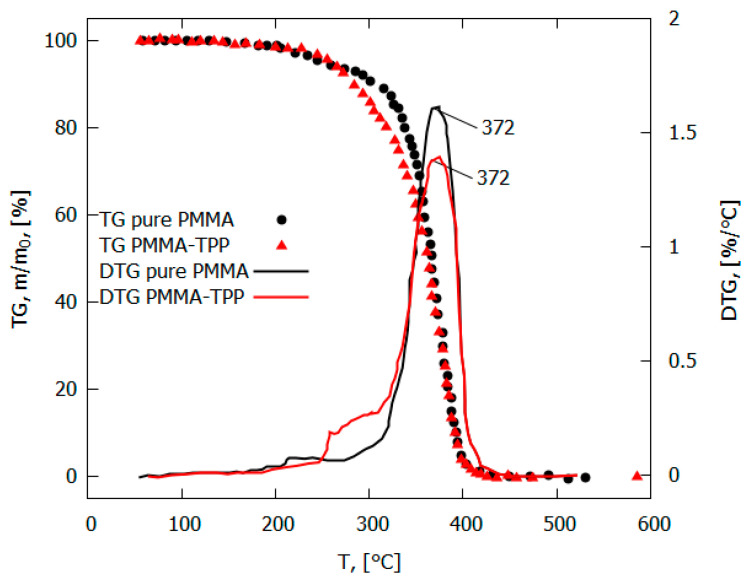
TGA and DTG of PMMA and PMMA-TPP in inert medium (10 K/min).

The addition of the flame-retardant TPP led to a decrease in the flame propagation rate, the mass combustion rate, the size of the pyrolysis zone, and the magnitude of the incident heat flux from the flame to the surface of the PMMA polymer [49,50] (Table 1). At the same time, no noticeable effect of TPP on the rate of the main stage of the thermal decomposition of PMMA was found [49]. The maximum rate of the decomposition of the PMMA and PMMA + 10% TPP samples in an inert medium corresponded to a temperature of 372 °C (Figure 9). The low-temperature stage for the PMMA + 10% TPP sample probably corresponds to the early release of TPP, which is favorable to the gas-phase mechanism of flame-retardant action, which enters the flame zone earlier.

In [49,50], the numerical simulation of flame propagation over PMMA and PMMA with the addition of TPP was carried out. To take into account the effect of the flame-retardant in the gas phase, it was assumed that the additive reduces the rate of the global macroreaction of MMA oxidation: MMA + O_2_ -> Products, in accordance with the following formula:

(15) $$W_{g}=k_{g}\left(1-\gamma Y_{\mathrm{TPP}}\right)Y_{F}Y_{O}\exp\left(-\frac{E_{g}}{R_{0}T}\right)$$

where $k_{g}$ is the pre-exponential factor of the gas-phase reaction rate (same for pure PMMA and PMMA-TPP), $Y_{\mathrm{TPP}}$ is the TPP inhibitor’s mass fraction in the solid phase, and γ is the inhibitor effect coefficient.

The rate of thermal decomposition was determined as:

(16) $$W_{s}={\left(1-\alpha \right)}^{n}k_{s}\exp(-E_{s}/R_{0}T)$$

where α is the conversion degree, *R*_0_ is the universal gas constant, and *T* is the temperature.

**Figure 10:**
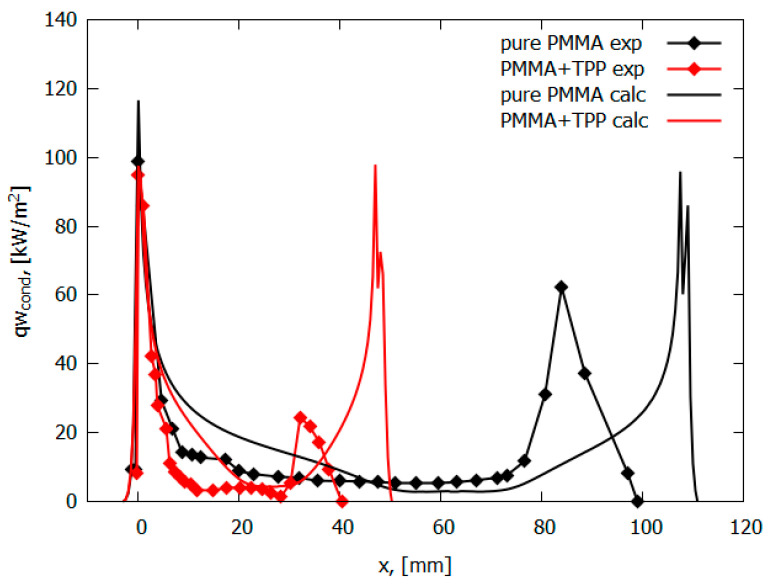
Conductive heat flux profiles for PMMA and PMMA + 10%TPP.

The measured conductive heat flux from a PMMA + 10% TPP flame for horizontal flame propagation decreases by a factor of 10 to 10 kW/m^2^ at a distance of 5 mm from the flame front. On the contrary, the conductive heat flux from the PMMA flame remains constant at the level of 15 kW/m^2^ almost until the end of the pyrolysis zone (Figure 10). For a horizontal flame spread over PMMA and PMMA + 10% TPP, it was assumed that the rate constant of the gas-phase reaction decreases by a factor of 50 (*γ* = 9.8) (Table 2). The parameters kg and γ in the model were chosen to agree with the experimental data on the flame propagation rate. The developed model, despite the simplifications used, well predicts the temperature profiles and heat flux near the leading flame front and satisfactorily predicts the second peak, which was small in the experiment due to an experimental error relating to the size of the pyrolysis zone (Figure 10). In addition, the model satisfactorily predicts the concentration distributions of the main species in the PMMA and PMMA + 10% TPP flames [49].

**Figure 11:**
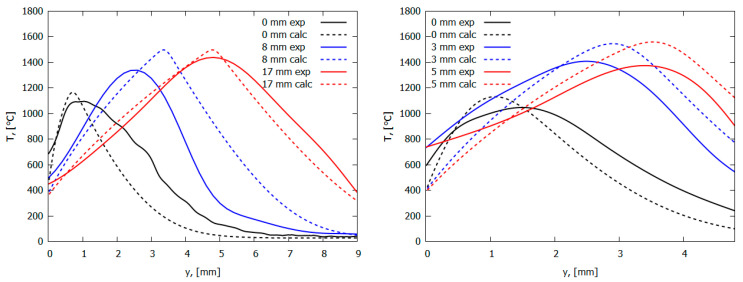
The temperature profiles in pure PMMA flame (**left**) and PMMA + 10%TPP flame (**right**) in the normal direction to the burning surface (legend shows the distance from the flame front). Solid lines—experiment; dashed lines—modeling.

The measured conductive heat flux (Figure 10) from the PMMA-TPP flame, obtained from the local vertical temperature profiles (Figure 11), is slightly less than that of the PMMA flame in the leading edge (Figure 10) The heat flux takes its maximum value near the flame front (at a distance of ~0 mm from the location of the maximum surface temperature). For PMMA without additives, the maximum heat flux is 99 ± 24 kW/m^2^; this value agrees satisfactorily with the simulation (116.6 kW/m^2^). In the case of PMMA + 10%TPP, the maximum heat flux is insignificantly lower than that of PMMA and equals 95 ± 24 kW/m^2^, while the predicted value is 97.9 kW/m^2^. Far from the flame front (where there is no oxygen near the surface), TPP decomposition products inhibit gas-phase combustion; the heat flux for PMMA + 10% TPP is less than that of PMMA.

A TPP molecule contains a phosphorus atom in its composition. In [50], using quantum chemical calculations, it was determined that in an inert medium (near the combustion surface), the main decomposition products of TPP are PO and PO_2_ radicals. These radicals are responsible for the recombination of H and OH radicals, leading to the termination of MMA oxidation chains in the flame.

**Figure 12:**
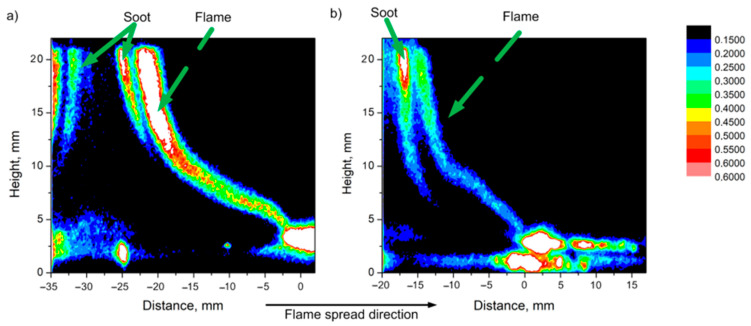
PLIF images (intensity corresponds to the concentration of OH radicals) of (**a**) PMMA flame and (**b**) PMMA + 10% TPP flame during horizontal flame propagation.

In [56], using the planar-induced fluorescence (PLIF) technique, it was shown that when TPP is added to the PMMA composition, a smaller amount of OH radicals is observed in a horizontally propagating flame. This is illustrated in Figure 12, which shows images of the intensity fields (I) of the OH radical fluorescence in a PMMA flame (left, a) and PMMA + 10% TPP (right, b). The areas with the brightest glow correspond to the flame front (the flame front position approximately corresponds to a distance of 0 mm in Figure 11). It can be seen from Figure 12 that, with the addition of 10% TPP, the intensity of PLIF-OH decreased by about 30%, and with the addition of 20% TPP, it decreased by about ~63%. Notably, the relative reduction in OH concentration roughly corresponds to the effect of the additive on the flame propagation rate (Table 1).

The data obtained testify in favor of the gas-phase mechanism of the effect of TPP on the flammability of PMMA during horizontal flame propagation. During the combustion of PMMA and PMMA with flame-retardant additives, ~99% of the initial fuel enters the gas phase; i.e., the formation of a carbon frame does not occur. An elemental analysis of the soot formed during the combustion of PMMA samples with a flame-retardant additive of 20% TPP (phosphorus content in the sample 1.89%), carried out in [56], showed that the amount of phosphorus in the soot was 0.3% of the initial mass of the sample. Consequently, most of the phosphorus passed into the gas phase, and a smaller part was involved in the process of soot formation.

**Figure 13:**
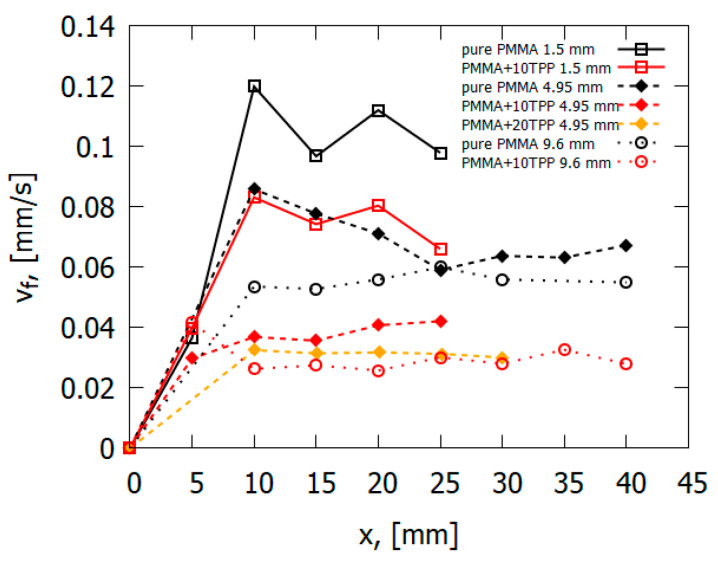
The downward flame-spread rate as a function of the distance from the slab edge.

**Figure 14:**
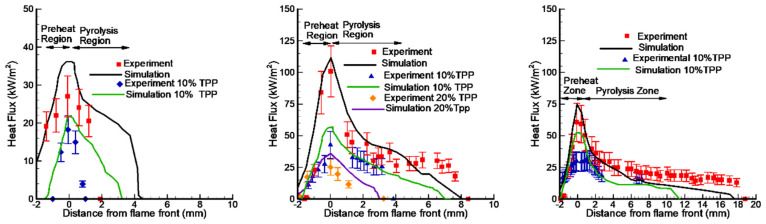
Comparison of the experimental and numerical distribution of conductive heat flux across the combustion zone of PMMA slabs of 1.5 mm, 4.95 mm, and 9.6 mm thicknesses without TPP (red symbols) and with 10%TPP (blue symbols) and 20%TPP (orange symbols).

The data obtained during the flame spread over the vertical PMMA slab with and without the addition of TPP also testify in favor of the gas-phase mechanism of TPP action [50]. Table 1 and Figure 13 illustrate the experimentally determined flame spread rates for samples with and without TPP as a function of the distance from the upper edge (the ignition point) of the sample. As 10% TPP was added to 1.5 and 4.95 mm-thick PMMA slabs, ~30–35% and 50–60% reduction was observed in the steady-state flame spread rates, correspondingly. When TPP was increased to 20% in the 4.95 mm sample, the flame spread rate further decreased; however, the difference is not as significant as the difference between the results for samples with 0% and 10% TPP additions in this case. Furthermore, the addition of the flame-retardant results in a decrease in the width of the flame zone, the size of the pyrolysis zone, and the heat flux from the flame to the combustion surface (Figure 14), which leads to a decrease in the rate of the flame’s spread (Figure 13).

A comparison of the experimental data [50] and the numerical prediction of the conductive heat flux to the PMMA surface for the three fuel thickness values (1.5 mm, 4.95 mm, and 9.6 mm) for samples without and with 10% and 20% TPP additives (Figure 14) shows their satisfactory agreement. With the addition of TPP flame-retardant, a decrease in the value of the incident heat flux was observed, which led to a decrease in the rate of flame propagation.

**Figure 15:**
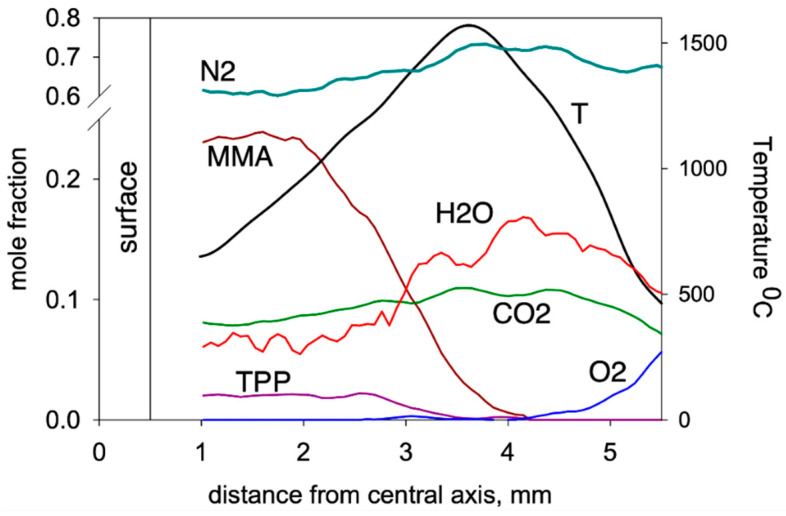
Species concentration and temperature profiles in the flame of a downward 90% PMMA + 10% TPP slab (4.6 mm thick) at a height of 3 mm from the flame front.

Using the molecular-beam sampling method (MBMS), the structure of the downward spreading flame over the PMMA + 10%TPP slab was measured [50]. Figure 15 presents the temperature profiles and the concentration profiles of the main combustion products (MMA, O_2_, CO_2_, H_2_O, TPP). MMA was consumed at a distance just short of 2 mm from the sample’s centerline. At this point, the temperature was ~950 °C, and TPP began to be consumed significantly at a higher temperature (~1150 °C) and at a longer (>2.7 mm) distance. The complete consumption of MMA and TPP occurred in the absence of oxygen at a distance of ~3.5 mm before the temperature maximum.

Mass peaks corresponding to species such as C_6_H_6_, HOPO_2_, HOPO, PO_2_, and PO were also detected [50], and their intensities were measured considering the contributions of the fragment ion peaks from TPP. Their presence suggests that they are involved in recombination reactions of H and OH radicals according to the mechanism [68].

Subsequent reactions involving the above radicals can lead to the formation of HOPO and HOPO_2_, which are responsible for the inhibition of MMA oxidation. This indicates the correctness of the assumption about the gas-phase mechanism of action of TPP as a flame-retardant. The effect of the TPP additive on the combustion process is taken into account in the model with a relatively simple decrease in the rate constant of the gas-phase macroreaction of MMA oxidation in the flame (Table 2). The PO and PO_2_ radicals formed during the thermal decomposition of TPP participate in recombination reactions with H and OH radicals (4), leading to a decrease in the concentration of the latter [50]. The obtained results of numerical simulation confirm the proposed mechanism of the effect of TPP on the combustibility of PMMA. It can be concluded from the data obtained that the gas phase is the main site of action of TPP as a flame-retardant, and the inhibition of fuel oxidation processes in the flame—due to quenching radicals in the reactions caused by their recombination with organophosphorus compounds—serves as the main mechanism of action.

### 4.2. The Mechanism of Action of TPP on the Thermal Decomposition and Combustion of PE

Studies on the effect of TPP addition on the combustion of polymers from the polyolefin class are presented in a series of works [60,61,62,69,70,71,72,73], in which polyethylene with various molecular weights was studied as a polymer. Table 3 presents the data available in the literature on the combustion and pyrolysis of both pure polyethylene and its blends with TPP.

#### 4.2.1. The Effect of TPP Addition to UHMWPE and HDPE on the Burning Rate and LOI

The results of measuring the burning rate of UHMWPE (MW = 5 × 10^6^) and HDPE (MW = 3.8 × 10^5^ and 10^5^) samples with 10% TPP during their diffusion combustion in air in candle mode showed that this parameter noticeably decreases, and the decrease in the flame propagation velocity depends on the molecular weight of the polymer [60,69,70,71,72,73]. In the case of UHMWPE, the burning rate decreases by a factor of two, and for HDPE with MW = 3.8 × 10^5^ and 10^5^, this change is a factor of five and ten, respectively. Furthermore, the addition of 10% TPP resulted in an increase in LOI for UHMWPE (MW = 5 × 10^6^) and HDPE (MW = 3.8 × 10^5^ and 10^5^) by 20%, 13%, and 15%, respectively (Table 3). For the counterflow flame of UHMWPE (MW = 2.5 × 10^6^) in air, the addition of 5% TPP led to a decrease in the burning rate from 0.018 to 0.0077 mm/s, i.e., by ~57%, and also reduced the strain rate at the moment of flame extinction by ~28% [61,62].

Thus, according to the experimental data obtained, the combustibility of polyethylene samples with different molecular weights significantly decreased with the addition of TPP. These facts indicate only a decrease in the flammability of polyethylene as a whole, but do not allow us to reveal the mechanism and location of the action of the fire-retardant. Therefore, for a deeper understanding of the physicochemical processes that occur during the combustion of polyethylene with the addition of TPP, comprehensive studies of the pyrolysis kinetics and the thermal and chemical structures of the flames of polyethylene blends with TPP were carried out, the results of which are given below.

#### 4.2.2. Inhibition of UHMWPE and HDPE Pyrolysis in an Inert Medium

**Figure 16:**
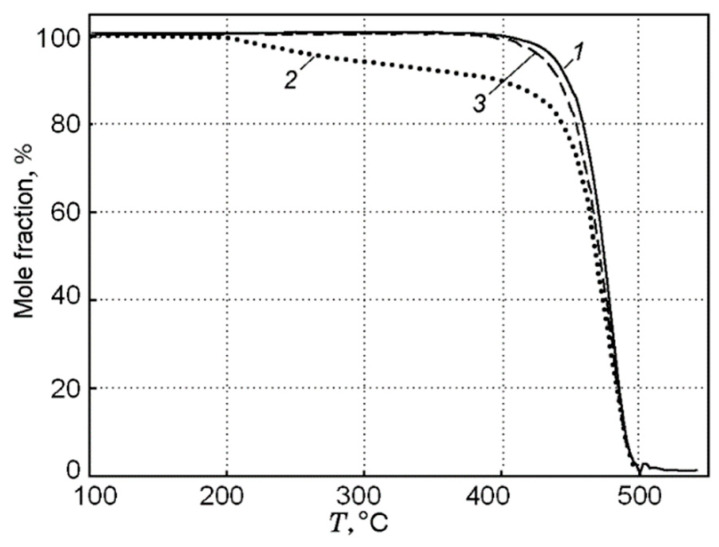
Thermogravimetric curves for UHMWPE (1) and UHMWPE + 10% TPP (2); Curve 3 was obtained from Curve 2 (see the text); the heating rate was 10 K/min.

A study of the pyrolysis of UHMWPE and UHMWPE + 10% TPP powders with the TGA method at a low heating rate of ~10 K/min made it possible to establish that, under these conditions, UHMWPE and TPP do not interact with each other [60,70]. This follows from the fact that during the pyrolysis of a blend of UHMWPE and TPP, a noticeable weight loss begins to be detected at a temperature T ≈ 200 °C (Figure 16), which corresponds to the boiling point of TPP. The dependence of weight loss (Curve 2 in Figure 16) has two characteristic stages: During the first stage (200–400 °C) the weight loss exactly corresponds to the proportion of TPP in the virgin sample. In the second stage (400 ÷ 500 °C), the remaining fraction (90%) of the initial mass of the blend decomposes. The result of normalizing 90% of the mass to 100% in the range of 400 ÷ 500 °C (obtained by multiplying by 100/90) presented in Curve 3 of Figure 16 shows that Curve 1 (100% UHMWPE) and Curve 3 almost coincide; thus, we can say that that only UHMWPE remained in the blend, and, therefore, at a low heating rate in an inert medium, UHMWPE and TPP do not interact with each other.

**Figure 17:**
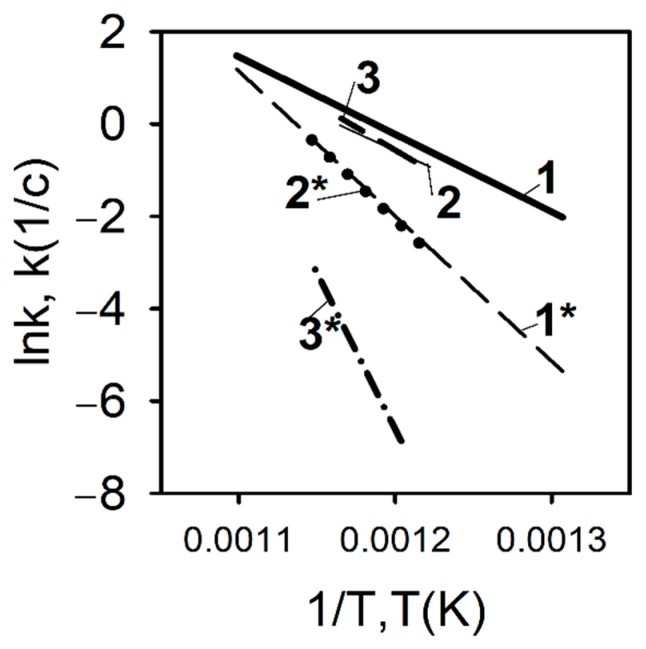
The rate constant of PE and PE/TPP (10%) degradation at a high heating rate (150 K/s). 1—UHMWPE (5 × 10^6^); 1*—UHMWPE (5 × 10^6^) + 10%TPP; 2—HDPE (3.8 × 10^5^); 2*—HDPE (3.8 × 10^5^) + 10% TPP; 3—HDPE (10^5^); 3*—HDPE (10^5^) + 10% TPP.

In contrast to the pyrolysis conditions of a polyethylene blend with TPP at a low heating rate, a completely different situation develops during pyrolysis under high-temperature heating conditions. Figure 17 shows the results of measuring the pyrolysis rate constants determined by the DMSTA method at a heating rate of 150 K/s for polyethylene samples with different molecular weights—10^5^, 3.8 × 10^5^, and 5 × 10^6^, without additive and with the addition of 10% TPP [60,70,71].

As can be seen from Figure 17, in contrast to pyrolysis at a low heating rate, with high-speed heating (150 K/s, Ar), a well-pronounced inhibition effect of TPP addition can be observed, which is most pronounced in the case of low-molecular-weight HDPE (MW = 10^5^). When TPP is added to UHMWPE and HDPE (3.8 × 10^5^), an increase in the activation energy of the decomposition reaction, by a factor of two, is observed (from 139 to 261 kJ/mol for UHMWPE; from 139 to 270 for HDPE (3.8 × 10^5^); and for HDPE (10^5^), three times (from 172 to 561 kJ/mol). Thus, it was experimentally shown in [60,70,71] that, at a high heating rate in an inert medium, TPP acts as an inhibitor in the condensed phase, and the effectiveness of the TPP additive on the pyrolysis of polyethylene increases when its molecular weight decreases.

At the same time, an interesting fact was discovered in [61] related to the effect of TPP on the average molecular weight of the gaseous products of UHMWPE pyrolysis. Using gas chromatographic analysis, it was found that, at a distance of 0.8 mm from the polymer surface, the addition of TPP reduces the average molecular weight of hydrocarbons from 258.7 g/mol (for pure UHMWPE) to 129.7 g/mol (for a blend of UHMWPE + 5% TPP). These data indicate a noticeable effect of TPP on the processes of the thermal decomposition of UHMWPE in the condensed phase.

#### 4.2.3. The Effect of TPP Addition on the Thermal and Chemical Structure of a Polyethylene Diffusion Flame in the “Candle” Mode

##### Temperature Profiles

**Figure 18:**
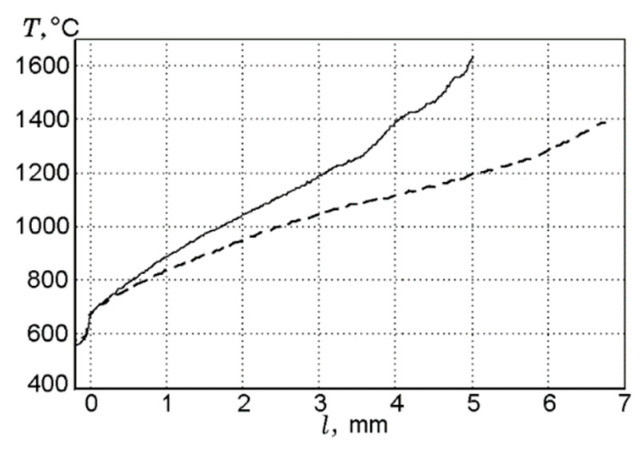
Temperature profiles in the diffusion flames of UHMWPE (solid curve) and UHMWPE + 10% TPP (dashed curve) in air.

In the case of combusting UHMWPE and UHMWPE + 10% TPP samples in air in the “candle” mode, the addition of TPP leads to a noticeable decrease in temperature in the combustion zone, which indicates the inhibitory effect of adding TPP [60,70]. Measurements of the temperature profiles in the diffusion flames of UHMWPE and UHMWPE + 10% TPP, using a Π-shaped thermocouple, showed that the addition of 10% TPP to UHMWPE reduced the temperature gradient in the gas phase in the reaction zone, but this had almost no effect on the surface temperature of the samples (Figure 18). Thus, in this figure, a well-pronounced effect of the addition of TPP in the gas phase can be observed.

##### Species Concentration Profiles

**Figure 19:**
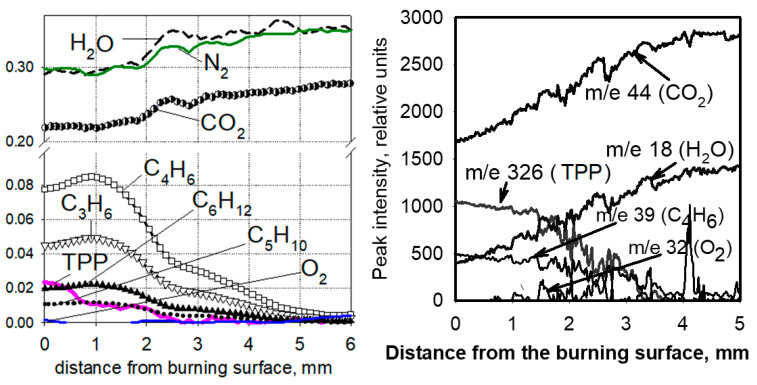
Species concentration profiles in the diffusion flame of samples of UHMWPE + 10% TPP (**left**) and HDPE (3.8 × 10^5^) + 10% TPP (**right**).

One of the important issues in understanding the mechanism of action of flame-retardants is information about whether the vapor of the flame-retardant or the products of its pyrolysis is released from the condensed phase into the gas phase, as well as in what ratio the flame-retardant is distributed between the gas phase and the condensed one. To clarify this issue, in [60,70,71,72], the structure of a diffusion “candle” flame of UHMWPE + 10% TPP and PE (3.8 × 10^5^) + 10% TPP in air was studied with molecular-beam mass spectrometry. A flame structure measurement was carried out along the central axis of the sample. Figure 19 shows the dependence of the mole fractions of the main identified species (N_2_, O_2_, CO_2_, H_2_O, TPP, propylene (C_3_H_6_), butadiene (C_4_H_6_), pentene (C_5_H_10_), and hexene (C_6_H_12_)) in a diffusion “candle” flame compared with the distance to the combustion surface of the sample. Oxygen is absent in this flame zone up to a distance of 5 mm from the polymer combustion surface, suggesting that the pyrolysis of TPP occurs in inert atmospheres. The zone of consumption of the pyrolysis products in the flame was 4–5 mm. In the gas phase, at a distance of 0–4 mm from the combustion surface, TPP vapors were found, indicating that during the combustion of UHMWPE + 10% TPP and HDPE (3.8 × 10^5^) + 10% TPP, a significant fraction of TPP (~40%) is released into the gas phase. This indicates that the place of action of the TPP in a blend with UHMWPE and HDPE is not only the condensed phase but also the gas phase.

#### 4.2.4. The Effect of TPP Addition on the Thermal and Chemical Structure of Polyethylene–Air Counterflow Flame

##### Temperature Profiles

**Figure 20:**
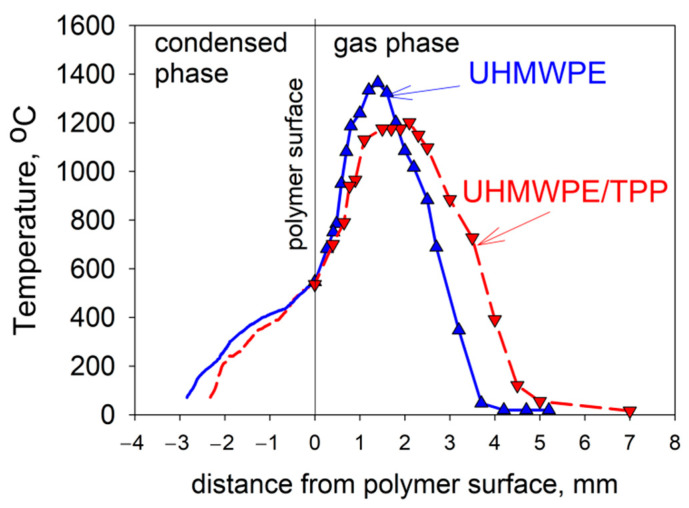
The temperature profiles in the condensed and gas phases in UHMWPE burning. Solid line—UHMWPE; dashed line—UHMWPE + 5 wt% of TPP.

Due to the physical features of HDPE with a molecular weight of MW = 10^5^ and 3.8 × 10^5^—namely, the formation of a large amount of molten polymer on the surface of the samples during their combustion—it was not possible to study the thermal and chemical structure of HDPE-air counterflow flame. However, in the case of studying UHMWPE with MW = 2.5 × 10^6^, this was successfully implemented [61,62,74]. The temperature profile measurements shown in Figure 20 indicate that the addition of 5% TPP to UHMWPE led to an increase in the total width of the flame zone by a factor of ~1.35 (from 3.7 mm to 5 mm), a shift in the temperature maximum point from 1.5 mm to 2.2 mm, and a decrease in the maximum temperature value by 150 °C (from 1380 °C to 1230 °C). In these experiments, the addition of TPP had almost no effect on the polymer surface temperature, which is 522 ± 8 °C. Thus, the data presented in Figure 20 indicate that the main effect of adding TPP appeared in the gas phase.

##### Species Concentration Profiles

**Figure 21:**
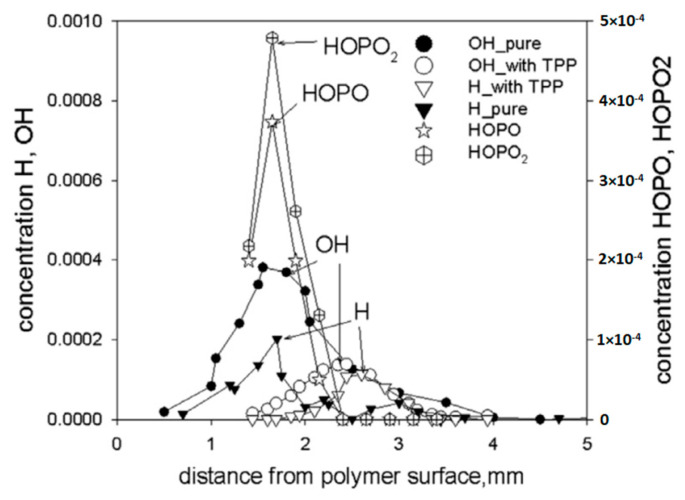
The concentration profiles of H, OH, HOPO, and HOPO_2_ in the flames of UHMWPE without and with 5 wt% TPP.

Using probing molecular-beam mass spectrometry with soft ionization, in [62,74,75], the chemical structure of the UHMWPE–air counterflow flame without an additive and with 5% TPP was measured. The main result of this study, which confirmed the gas-phase mechanism of action of the addition of TPP to UHMWPE, was a strong decrease in the maximum concentration of H and OH in the zone of basic chemical reactions when 5% TPP was added to the polymer. Figure 21 shows the profiles of the concentration of H and OH radicals in the UHMWPE–air counterflow flame without an additive and with the addition of TPP, from which it can be seen that the addition of TPP led to a decrease in the maximum concentration of H and OH by about two times, as well as a shift in their maxima by about 1 mm from the burning surface. Thus, it has been demonstrated by direct experiments that the effect of TPP consists in the participation of this species and its decomposition products in chain-termination reactions. Direct experimental evidence of this fact is also the measured concentration profiles of HOPO and HOPO_2_ in the flame of UHMWPE + 5% TPP (Figure 21). According to previous studies on the chemistry of the destruction of organophosphorus species, HOPO and HOPO_2_ are formed as the final products of the decomposition of these species in hydrogen and hydrocarbon flames [68], and due to the flow of catalytic cycles, we can see that:

H + PO_2_ + M→HOPO + M

HOPO + OH→PO_2_ + H_2_O

OH + PO_2_ + M→HOPO_2_ + M

HOPO_2_ + H→PO_2_ + H_2_O

It is they that cause the recombination of the main carriers of chain reactions; as a result, the rate of combustion processes decreases until the flame is completely extinguished.

Thus, the analysis of the entire dataset obtained showed that the reactions in the condensed and gas phases in the PE + TPP flame play opposite roles in the mechanism of reducing flammability. Adding TPP to PE inhibits its thermal decomposition in an inert atmosphere at high heating rates (~150 K/s) and has no effect at low heating rates (0.17 K/s). With a decrease in the molecular weight of PE, the inhibitory effect of the TPP additive increases, which also affects the burning rate of PE + TPP blends in the case of combustion in a “candle” mode. At the same time, the following additional facts were found, indicating that the action of TPP in the condensed phase can increase the flammability of UHMWPE, namely: the formation of phosphorus-containing compounds (phosphates, ethers, carbonates) on the surface of the UHMWPE + TPP sample, which leads to a decrease in the yield of TPP in the gas phase, where it effectively reduces the concentration of radicals; a twofold decrease in the molecular weight of heavy hydrocarbon products of the pyrolysis of a blend of UHMWPE + TPP (hydrocarbons with a lower molecular weight are oxidized at a higher rate); and an increase in the burning rate leads to an increase in the flow rate of decomposition products from the combustion surface of UHMWPE due to flame moving to the combustion surface and an increase in heat flux to the surface.

At the same time, the following facts obtained in this work testify to the significant contribution of the gas-phase mechanism of action of TPP additive to reducing the flammability of UHMWPE, namely: broadening the flame zone by 1.5 times and a decrease in the maximum flame temperature by 150 °C, leading to a decrease in the heat flux from the flame on the polymer surface, which was experimentally confirmed with direct measurements of the temperature gradients near the combustion surface; the reduction of the extinction strain rate in the UHMWPE–air counterflow flame by a factor of 1.5 when TPP is added; and a decrease in the maximum concentrations of H and OH radicals after the addition of TPP to UHMWPE, which is unequivocal evidence of the inhibitory effect of TPP on chain-branching reactions in the polymer flame.

Thus, although the action of TPP in the condensed phase of the polymer may result in a certain increase in its flammability, the effect of the action in the gas phase, leading to a decrease in flammability, is strongly dominant. This implies that the addition of 5–10% TPP is sufficient to markedly reduce the flammability of UHMWPE.

### 4.3. The Mechanism of Action of DOPO and Graphene on Flame Propagation over Glass-Fiber-Reinforced Epoxy Resin

The numerical model for reinforced material is close to the flame propagation model for PMMA. In both cases, the following are assumed: a non-stationary two-dimensional formulation of the problem, a quasi-stationary flame propagation mode, a coupled process of heat and mass transfer in a heterogeneous “gas–solid body” system, a one-stage gas-phase combustion reaction (Fuel + O_2_ -> Products) with a finite reaction rate in the form of the Arrhenius law, a one-stage pyrolysis reaction, and a laminar flow.

Based on the previous analysis of the experimental data, it was proposed that the main flame inhibition effect of composites and polymers containing flame-retardants based on DOPO takes place in the gas phase [76]. The pre-exponential factor of the gas phase combustion reaction is reduced according to Formula (14).

We considered the following when taking into account the action of a graphene-based FR. The experiment described in [65] demonstrated the intense formation of soot as flame spread over GFRER slabs, which contained FRs based on graphene. The soot became deposited on the thermocouples and the burning surface, which caused an increase in the porous layer on the slabs. Therefore, it was presumed that a non-combustible gas is part of the total gaseous pyrolysate, which is further converted into soot, while the other part goes to the gaseous fuel.

**Figure 22:**
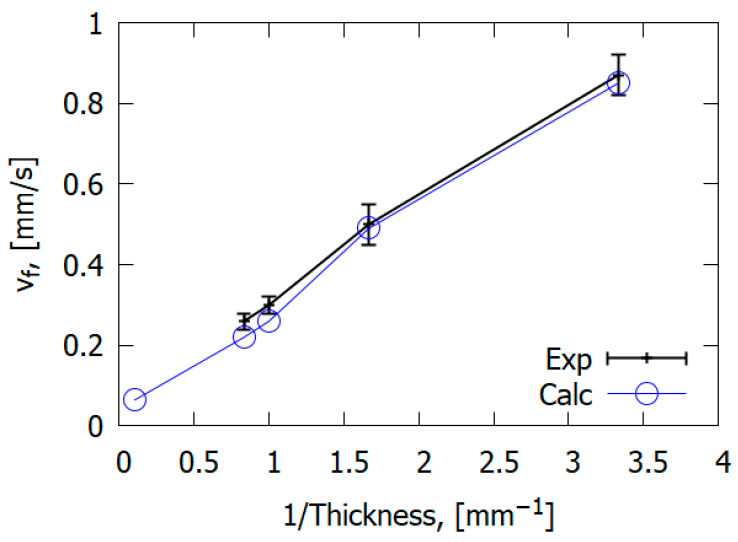
Dependence of the ROS over GFRER slabs on slab thickness. Exp—experiment; Calc—calculation.

The combustion mechanism of reinforced polymeric materials differs from the combustion of homogeneous polymers such as PMMA. However, general patterns can be observed in the case of glass-fiber-reinforced materials and non-reinforced polymers. For example, the study carried out by [67] showed that the downward ROS for glass–epoxy is inversely proportional to slab thickness (Figure 22), similarly to PMMA [50].

**Figure 23:**
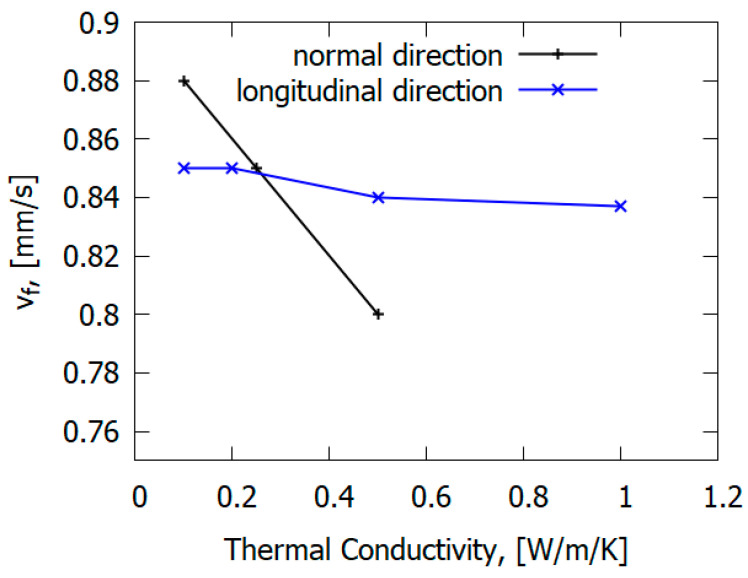
The effect of thermal conductivity in the longitudinal direction and in the normal direction on the flame spread rate over GFRER.

The main mechanism of flame propagation over glass–epoxy is the heating of solid fuel by the heat flux from the flame. However, the type of reinforcing fiber can affect the flame propagation mechanism. In contrast to carbon fiber plastics [77], in the case of glass fiber plastics (for GFRER), the ROS does not depend on the thermal conductivity along the glass–epoxy, but rather, it depends on the thermal conductivity in the direction perpendicular to the flame propagation velocity [67] (Figure 23). Thermal conduction in the normal direction to the slab has a greater effect on the rate of flame spread (ROS) than along it [67]. This is due to the properties of fiberglass, delaying the escape of heat in the direction of the fibers. For carbon-fiber-reinforced plastics [78], in contrast to fiberglass plastics, preheating in the condensed phase along the direction of flame propagation has a stronger effect on the ROS due to the thermal conductivity of carbon fiber.

Since flame propagation for fiberglass plastics depends on the heat flux from the gas phase and the heat–gas exchange between the gas and condensed phase, the addition of flame-retardants should significantly affect the gas phase or both phases. In [65], the following flame-retardants were chosen: DDM-DOPO [79] and graphene [80].

The addition of 6% graphene flame-retardant led to a 1.5% rise in the LOI, and the addition of 6% DDM-DOPO flame-retardant brought about a 4.1% growth compared with the sample without an additive (Table 4). All the samples, including the pure sample, met the requirements of the UL-94 HB test. Adding an FR decreased the ROS in the UL-94 HB test, and adding DDM-DOPO caused the sample’s self-extinction. In the UL-94 HB test, graphene and DDM-DOPO added to the composite caused a reduction in the burning rates of the GFRER samples, and in the VBB test [66], it brought about a drop in the mass loss rate. In accordance with the results of the LOI and UL-94 HB tests, 6% DDM-DOPO proved to be more effective in reducing the flammability of the glass-fiber-reinforced polymer than the 6% graphene additive. In the VBB test, graphene and DDM-DOPO, exposed permanently to the pilot flame, reduced the mass loss rates of the samples similarly well. However, the total mass loss of the samples turned out to be greater in the samples containing an FR

**Figure 24:**
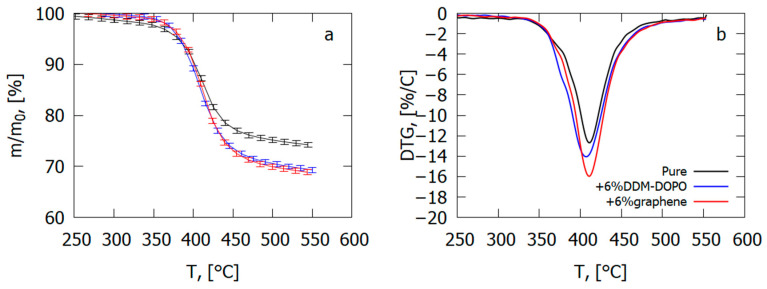
Thermal analysis data ((**a**)—TG, (**b**)—DTG) for GFRER composites with the addition of 6% DOPO-DDM or 6% graphene and without it.

In an inert medium, adding 6% graphene results in an increase in the thermal degradation velocity of the GFRER sample by 23% and an increase in the total yield of the volatile pyrolysis products by 4–5% (Figure 24), which correlates with the results of the VBB test (Table 4). The presence of 6% DDM-DOPO in the composite caused a 5K fall in the temperature of the degradation rate maximum and, thus, a rise in the yield rate of the volatile products of combustion in comparison with the pure sample (Table 4). It was found from the data on polymer degradation in air [65] that, in the case of GFRER without flame-retardant, the yield of char was ~9%. When flame-retardants were added, the total yield of the volatile pyrolysis products rose by 4–5% due to a decrease in the mass of the char formed.

Assuming a one-stage, first-order pyrolysis reaction (16), we obtained the kinetic parameters of pyrolysis by employing a well-established method [81], presented in Table 5. These data were used to model flame propagation.

The influence of DDM-DOPO on the rate of thermal decomposition is small, while graphene influences the pyrolysis kinetics (Table 5). To agree with the experimental data, it was assumed that DDM-DOPO reduces the rate constant of the global gas-phase combustion reaction by a factor of 1000, acting as a gas-phase flame-retardant. The addition of graphene leads to the fact that 30% of the pyrolysis products are non-combustible, and 70% participate in the combustion reaction. Therefore, graphene acts in both the gas and condensed phases.

Glass-fiber-reinforced epoxy resin does not support combustion in air (LOI > 22.4, Table 4). Therefore, flame propagation over reinforced composites, especially with the addition of flame-retardants, was studied under external flame conditions [66] or under the forced flow of an N_2_/O_2_ mixture with an increased concentration of O_2_ [78].

**Figure 25:**
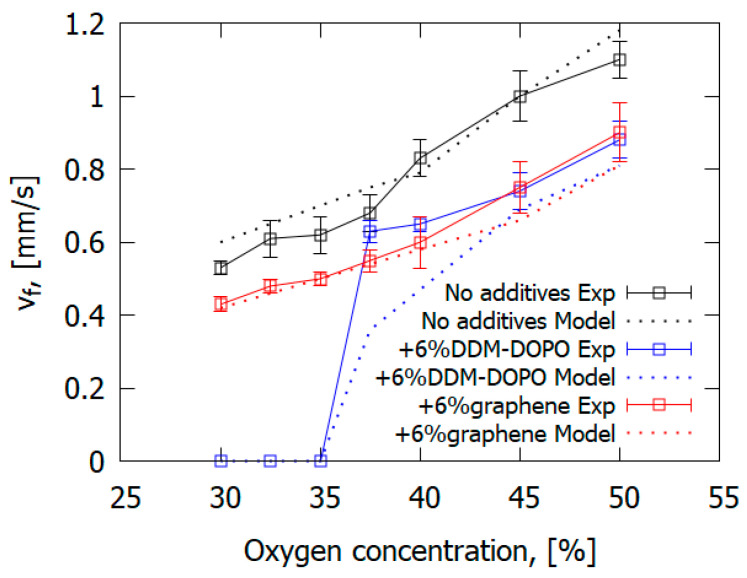
The measured and simulated dependences of the ROS on the O_2_ concentration for downward flame spread over GFRER slabs.

The presence of 6% graphene or 6% DOPO-DDM in the composite resulted in a decreased ROS over GFRER in the opposed flow of N_2_/O_2_ (Figure 25 and Table 6). For all O_2_ concentrations used, adding the flame-retardant resulted in a decrease in the ROS. However, the effect of flame-retardants and oxygen concentration in the opposed flow on the maximum surface temperature in the flame front was small or absent. The model satisfactorily predicted the surface temperature and the ROS. The surface temperatures in the model and experiment were compared in the preheating region and in the flame front since, because of the formation of soot far from the flame front, the temperature measurements with the IR camera caused an error due to a possible change in the surface emissivity.

As the O_2_ concentration was 35%, the GFRER + 6% DDM-DOPO slabs self-extinguished in the experiment; whereas, for the GFRER and GFRER + 6% graphene samples, self-sustained burning was recorded. Thus, the GFRER + 6% DDM-DOPO sample had the highest LOI among the studied samples, as well as the highest limiting oxygen concentration (LOC), equal to 35%. Due to the presence of phosphorus in DDM-DOPO, it was presumed that the degradation products of DDM-DOPO formed in the gas phase participated in the recombination reactions of H and OH radicals, causing flame extinction, similar to the action of TPP [50,56]. In the experiment, the effects of both FRs on the decrease in the rate of flame spread at O_2_ concentrations higher than 37.5% were actually the same, which demonstrated conformance with the results of the VBB test. That is, the effect of DDM-DOPO is close to that of graphene with the same additive concentration, but DDM-DOPO reduces the LOC for igniting GFRER.

The computer simulation results demonstrated in Figure 25 predicted the change in the ROS depending on O_2_ concentration quite well, both for the pure sample and for the samples containing graphene and DDM-DOPO additives. In the computer simulation, the self-extinction of the sample containing 6% DDM-DOPO additive occurred at 35% oxygen concentration (LOC) and lower. Similar to the above, the self-extinction of the samples was observed in the experiment, which can be explained by the insufficient heat release in the gas phase in the case of the self-sustained flame spread. The model proved to be a good predictor of the dependence of the ROS on O_2_ concentration for slabs containing graphene, decreasing the amount of the combustible pyrolysis products, which took part in the combustion reactions and increased the amount of non-combustible pyrolysis products (gaseous soot).

**Figure 26:**
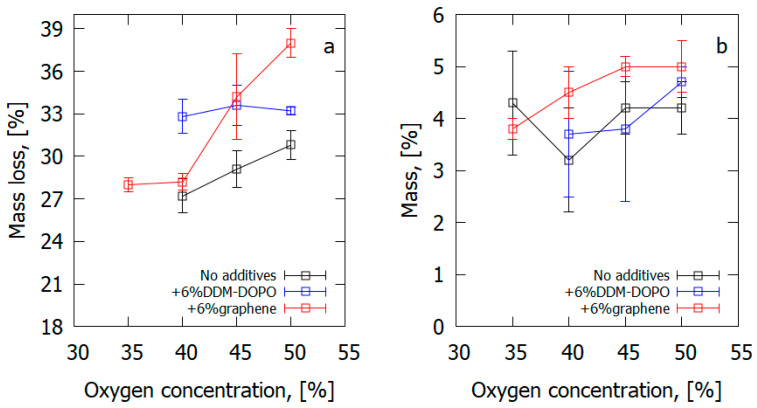
(**a**) The measured total mass loss of a sample after the experiment and (**b**) the mass of soot collected from the sample surface after the experiment.

Thus, the increase in the oxygen concentration resulted in a rise in the amount of formed soot in the case of GFRER composites containing FRs (Figure 26). A graphene additive contributed to soot formation, especially at higher oxygen concentrations. At high oxygen concentrations (>40% O_2_), the mass loss of the sample proved to be greater in the FR-containing GFRER samples than in the uninhibited GFRER samples, which conformed with the TGA data. This fact was beneficial for the fire protection properties of FR because of the rising rate of the FR released onto the surface of the slab and in the gas phase.

Thus, it seems to be likely that graphene moved to the slabs’ surface and promoted the formation of soot there. The gaseous products of glass-fiber-reinforced composite degradation left the solid substance, but a certain portion of them did not participate in the oxidation process and instead accumulated on the slab surface as soot. It is also probable that graphene promoted the condensation of the resin decomposition products, causing the formation of less burnt fuel in the gas phase and, correspondingly, lower heat flux from the flame on the polymer surface. In turn, the less intense heat flux led to a decreased ROS.

The future development of probe and non-contact methods for diagnosing flames, as well as the development of detailed models of flame propagation over polymers with flame-retardant additives, will improve our understanding of the mechanism of the effect of flame-retardants on the flammability of polymeric materials, which is an important task from the point of view of fundamental science and the development of effective fire prevention methods.

## 5. Conclusions

In the present review, using an integrated approach based on the experimental and theoretical study of the processes of the thermal decomposition and combustion of materials that are practically important polymers, such as polymethyl methacrylate, polyethylene, and glass-fiber-reinforced epoxy resin, we identified the features of the mechanism of reducing the combustibility of these materials when phosphorus-containing flame-retardant additives are introduced to them, as well as graphene.

A set of original experimental methods was developed and applied, which make it possible to study the kinetics of thermal decomposition and the thermal and chemical structure of the flames of the studied polymeric materials, including those with additives of fire-retardants, as well as to measure the flame propagation velocity, the mass burning velocity, and the values of heat fluxes from the flame to the surface of condensed materials. The developed methods can also be used to study the mechanism of action of various flame-retardants in other polymeric materials.

Numerical models were developed and tested to describe the key parameters of the flames of the studied polymeric materials, including those involving the addition of phosphorus-containing flame-retardants and graphene, such as the thermal and chemical structure of the flames, flame propagation velocity, the mass loss rate, the intensity of radiation, and the total heat flux on the surface of the condensed material, as well as the effect of the concentration of the flame-retardant additive on these parameters and the composition and velocity of airflow.

An analysis of the experimental data presented in the review, and the results of the numerical simulation for various polymeric materials—PMMA, polyethylene, and glass-fiber-reinforced epoxy resin—showed that the main effect of phosphorus-containing fire-retardants (TPP and DOPO) on reducing the combustibility of these materials is associated with inhibiting combustion processes in the gas phase, and the effect of adding graphene manifests itself in both the gas and condensed phases.

## Figures and Tables

**Table 1:** The main flame propagation characteristics.

Sample	TPP, wt%	Thickness, mm	Flame Propagation Direction	ROS, mm/s	Mass Loss Rate, g/s	Pyrolysis Length, L_p_, mm	LOI	Ref.
PMMA	0	1.5	downward	0.11	0.017	3	-	[50]
PMMA + 10%TPP	10	1.5	downward	0.073 (−33%)	0.007	1	-	[50]
PMMA	0	4.95	downward	0.065	0.022	9	17	[50]
PMMA + 10% TPP	10	4.95	downward	0.04 (−38%)	0.01	4	-	[50]
PMMA + 20% TPP	20	4.95	downward	0.03 (−54%)	0.01	5	-	[50]
PMMA	0	9.6	downward	0.055	0.062	18	-	[50]
PMMA + 10% TPP	10	9.6	downward	0.03 (−45%)	0.018	9	-	[50]
PMMA	0	5	horizontal	0.12	0.078	90	17	[49]
PMMA + 10% TPP	10	5	horizontal	0.075 (−37%)	0.054	35	21	[49]

**Table 2:** Kinetic parameters of pyrolysis and combustion macroreaction rates in the model (horizontal flame spread [49]).

	ROS, mm/s	$k_{s}$, 1/s	$E_{s}$, kJ/mole	k_g_ (1 − *γY*_TPP_), 1/s	Eg, kJ/mole
PMMA	0.12	6.45 × 10^7^	121.9	6 × 10^9^	90
PMMA + 10%TPP	0.075	2.29 × 10^10^	153.6	1.2 × 10^8^	90

**Table 3:** Characteristics of combustion and thermal decomposition of polyethylene of various molecular weights without additives and with the addition of TPP.

Sample	Molecular Weight of the Polymer	Fraction of Flame-Retardant, %	Direction of Flame Propagation	ROS, mm/s	ESR, 1/s	LOI	Peak Conc. H/OH, Mole Fraction	Pyrolysis Kinetics at a Heating Rate of 150 K/s in Ar, log (k_0_, (1/s)); E_a_, kJ/mole, Reaction Order	Maximum Flame Temperature, °C	Ref
UHMWPE	2.5 × 10^6^	0	counterflow	0.018	140		2 × 10^−4^/ 4 × 10^−4^		1380	[61,62]
UHMWPE + 5%TPP	2.5 × 10^6^	5	counterflow	0.0077 (−57%)	100 (−28%)		10^−4^/ 1.3 × 10^−4^ (−50%/ −68%)		1230	[61,62]
UHMWPE	5 × 10^6^	0	“candle”	0.1(0.25)				16.8; 249 2		[69,70]
UHMWPE + 5% TPP	5 × 10^6^	5	“candle”	0.04(0.11) (−60%)						[70]
UHMWPE + 10% TPP	5 × 10^6^	10	“candle”					20.5; 340; 2		[69,70]
UHMWPE	5 × 10^6^	0	“candle”	0.18		16.4		8.6; 139; 1		[60,72,73]
UHMWPE + 10% TPP	5 × 10^6^	10	“candle”	0.09 (−50%)		19.7 (+20%)		15.5; 261; 1		[60,72,73]
HDPE	3.8 × 10^5^	0	“candle”	0.22		17.6		7.9; 139; 1		[71,72]
HDPE + 10% TPP	3.8 × 10^5^	10	“candle”	0.04 (−80%)		19.9 (+13%)		16; 270; 1		[71,72]
HDPE	10^5^	0	“candle”	0.3		17.5		10.1; 172; 1		[71,72]
HDPE + 10% TPP	10^5^	10	“candle”	0.03 (−90%)		20.1 (+15%)		32.4; 561; 1		[71,72]

“candle”—burning in air with natural convection. ROS—speed of flame propagation through condensed matter. ESR—strain rate at flame extinction.

**Table 4:** The values of LOI, UL-94, and TGA; the mean mass loss rate ($\dot{m}$); and the total mass loss (TML) in the VBB test.

Sample	LOI, %	UL-94 HB, mm/min	$\dot{m}$ (VBB), g/s	Mass Loss (VBB), g	T_max_ DTG ^2^, °C
GFRER	22.4	44.5, burned-out ^1^	0.015 ± 0.002	0.51 ± 0.11	412
GFRER + 6% graphene	23.9	38.3, burned-out ^1^	0.010 ± 0.002	0.93 ± 0.18	411
GFRER + 6% DDM-DOPO	26.5	34.9, self-extinguished ^1^	0.010 ± 0.002	0.66 ± 0.14	407

^1^ HB rating; ^2^ temperature of the maximum decomposition rate in the TG test in an inert atmosphere (heating rate of 30 K/min).

**Table 5:** Kinetic parameters of pyrolysis and rates of gas-phase combustion macroreaction used in the simulation.

Sample	k_s_, 1/s	E_s_, kJ/mole	n	k_g_, 1/s	E_g_, kJ/mole	*ψ_DOPO_*	(1 − Y_F,s_), Non-Combustible Part
GFRER	6.97 × 10^10^	166	1	10^10^	90	0	0
GFRER + 6% graphene	1.4 × 10^19^	267	3	10^7^	90	0	0.3
GFRER + 6% DDM-DOPO	6.97 × 10^10^	166	1	10^10^	90	16.65	0

**Table 6:** The rate of flame spread (mm/s) over fiberglass-reinforced composites. The values in parentheses indicate the maximum surface temperature in the model and experiment in the flame front.

Oxygen Concentration	GFRER, Exp	GFRER + 6% Graphene, Exp	GFRER + 6% DDM-DOPO, Exp	GFRER, Model	GFRER + 6% Graphene, Model	GFRER + 6% DDM-DOPO, Model
30%	0.53	0.43	Not burned	0.6	0.42	0
32.5%	0.61	0.48	Not burned	0.65	0.46	0
35%	0.62	0.5	Not burned	0.7 (529 °C)	0.5	0
37.5%	0.68	0.55	0.63	0.75	0.54	0.36
40%	0.83 (~500 °C)	0.6 (~500 °C)	0.65 (~550 °C)	0.79 (544 °C)	0.58 (509 °C)	0.47 (549 °C)
45%	1 (~500 °C)	0.75 (~500 °C)	0.74 (~550 °C)	1 (557 °C)	0.66	0.69
50%	1.1 (~500 °C)	0.9 (~500 °C)	0.88 (~550 °C)	1.18 (549 °C)	0.81 (529 °C)	0.81 (556 °C)

## Abbreviations

AHP	aluminum hypophosphite
AlPi	aluminum diethylphosphinate
APP	ammonium polyphosphate
ATH	aluminum hydroxide
CF	carbon fiber
DDM-DOPO	4,4′-diamino-diphenylmethane 9,10-dihydro-9-oxa-10-phosphaphenanthrene-10-oxide
DMSTA	dynamic mass spectrometric thermal analysis
DOPI	heterocyclic 9,10-dihydro-9-oxy-10-phosphaphenanthrene-10-oxide tris-[(acryloyloxy)ethyl] isocyanurate
DOPO	9,10-dehydro,9-oxa,10-phosphophenanthrene,10-oxide
DOPP	9,10-dihydro-9-oxy-10-phosphaphenanthrene-10-oxide tetra-[(acryloyloxy)ethyl] pentarythrit
DSC	differential scanning calorimetry
DTG	differential thermogravimetric analysis
EG	expandable graphite
FR	fire-retardant
FTIR	Fourier transform infrared spectroscopy
GFRER	glass fiber reinforced epoxy resin
HDPE	high density polyethylene
HRR	heat release rate
IR	infrared
LDPE	low-density polyethylene
LOI	limiting oxygen index
MMA	methyl methacrylate
MRP	microencapsulated red phosphorus
PE	polyethylene
PLIF	planar laser-induced fluorescence
PMMA	polymethylmethacrylate
ROS	rate of flame spread
RP	red phosphorus
TD	triazine derivative
TGA	thermogravimetric analysis
TPP	triphenyl phosphate
UHMWPE	ultra-high-molecular-weight polyethylene
UL-94	standard for safety of flammability of plastic
VBB	vertical Bunsen burner test

## Nomenclature

C	specific heat capacity [J/kg/K]
D	diffusion coefficient [m^2^/s]
E	activation energy [J/mol]
g	gravity acceleration [m/s^2^]
k	preexponential factor [s^−1^]
M	molar mass [g/mol]
$\dot{m}$	mass burning rate [g/s]
n	pyrolysis reaction order [-]
p	pressure [Pa]
Q	heat release [J/kg]
q	heat flux [W/m^2^]
$R_{0}$	universal gas constant [J/mol/K]
T	temperature [K]
t	time [s]
u	velocity [m/s]
W	reaction rate [s^−1^]
x	coordinate along fuel surface [m]
Y	gas component mass fraction [-]
y	coordinate normal to fuel surface [m]
**Greek symbols**	
α	conversion degree [-]
γ	solid component mass fraction [-]
δ	burnout degree [-]
η	solid component volume fraction [-]
λ	thermal conductivity [W/m/K]
μ	dynamic molecular viscosity [kg/m/s]
ν	stoichiometric coefficient [-]
ρ	density [kg/m^3^]
τ	half-thickness [m]
Ψ	inhibition effect coefficient
**Subscripts**	
a	ambient
b	binder
F	fuel
F	fiber
g	gas
I	inert component
O	oxidizer
P	product
p	pyrolysis
s	solid
**Superscripts**	
r	radiative
x	along fuel surface
y	normal to fuel surface
